# Quantifying Overdiagnosis for Multicancer Detection Tests: A Novel Method

**DOI:** 10.1002/sim.10285

**Published:** 2024-11-26

**Authors:** Stuart G. Baker

**Affiliations:** ^1^ Division of Cancer Prevention National Cancer Institute Bethesda Maryland USA

**Keywords:** cancer screening, lead time, multicancer early detection test, overdiagnosis

## Abstract

Multicancer detection (MCD) tests use blood specimens to detect preclinical cancers. A major concern is overdiagnosis, the detection of preclinical cancer on screening that would not have developed into symptomatic cancer in the absence of screening. Because overdiagnosis can lead to unnecessary and harmful treatments, its quantification is important. A key metric is the screen overdiagnosis fraction (SOF), the probability of overdiagnosis at screen detection. Estimating SOF is notoriously difficult because overdiagnosis is not observed. This estimation is more challenging with MCD tests because short‐term results are needed as the technology is rapidly changing. To estimate average SOF for a program of yearly MCD tests, I introduce a novel method that requires at least two yearly MCD tests given to persons having a wide range of ages and applies only to cancers for which there is no conventional screening. The method assumes an exponential distribution for the sojourn time in an operational screen‐detectable preclinical cancer (OPC) state, defined as once screen‐detectable (positive screen and work‐up), always screen‐detectable. Because this assumption appears in only one term in the SOF formula, the results are robust to violations of the assumption. An SOF plot graphs average SOF versus mean sojourn time. With lung cancer screening data and synthetic data, SOF plots distinguished small from moderate levels of SOF. With its unique set of assumptions, the SOF plot would complement other modeling approaches for estimating SOF once sufficient short‐term observational data on MCD tests become available.

## 1 Introduction

The goal of cancer screening is to detect preclinical cancers, asymptomatic changes thought to indicate carcinogenesis, with the hope that early intervention will reduce cancer mortality. Multicancer detection (MCD) tests based on blood or urine specimens have the potential to detect preclinical cancer in many cancers for which there is no conventional screening technology. These technologies typically involve abnormal methylation, fragmentation patterns, mutations in cell‐free DNA, and protein markers in exosomes [1].

A major concern with cancer screening and particularly MCD tests is overdiagnosis, the screen–detection of a preclinical cancer that would not have developed into symptomatic cancer in the absence of screening [1, 2]. I define screen detection as a positive screening test followed by a work‐up that indicates preclinical cancer. My focus is overdiagnosis for specific cancers in an MCD test.

Because overdiagnosis can lead to unnecessary and harmful treatment, quantifying the amount of overdiagnosis is important. Two metrics for quantifying overdiagnosisare (i) the screen overdiagnosis fraction (SOF), the probability of overdiagnosis at screen detection, and (ii) the screen‐interval overdiagnosis fraction (SIOF), the probability of overdiagnosis among all cancers diagnosed during a cancer screening program [3]. For interpreting results, SOF is preferable to SIOF.

Quantifying overdiagnosis is “notoriously difficult” [4] because overdiagnosis is not directly observed. Estimating SOF with MCD tests is particularly challenging because short‐term results are needed as the technology is rapidly evolving. I introduce a novel method to estimate SOF for MCD tests using short‐term observational data. Because MCD tests are starting use in clinical practical, such data may become available soon. The requirements for this novel method are at least two yearly MCD tests for persons having a wide range of ages and a restriction to cancers for which there is no conventional screening.

The paper is organized as follows. Section 2 reviews commonly used methods for quantifying overdiagnosis in terms of their potential application to short‐term observational data from MCD tests. Section 3 introduces the novel method for quantifying overdiagnosis with short‐term observational data from MCD tests. Section 4 illustrates the novel method with real and synthetic screening data. Section 5 is a discussion.

## 2 Review of Methods to Quantify Overdiagnosis

Methods for estimating SOF or SIOF include cumulative excess incidence in a stop‐screen trial, a population trend analysis, and mathematical modeling.

### 2.1 Stop‐Screen Trial

In a stop‐screen trial, which is a randomized trial of screening versus no screening with follow‐up after the last screen, the estimated SOF is the cumulative excess cancer incidence in the screened versus the unscreened group divided by the cumulative number of screen‐detected cancers. The estimated SOF has negligible bias with a sufficiently long follow‐up time [5], but confidence intervals are wide [3]. A drawback to applying this method to MCD tests is the long time needed for unbiased estimation.

### 2.2 Population Trend Analysis

A population trend analysis estimates SIOF as the difference between the population yearly cancer incidence and the projected yearly background cancer incidence if there were no screening, divided by the yearly cancer incidence [6]. The long time needed to reach steady state after the introduction of population screening [7] precludes using this method for MCD tests.

### 2.3 Mathematical Models

Mathematical models of cancer progression during screening are the best strategy to estimate SOF from short‐term data. In many models, a person begins in a disease‐free state and may enter a screen‐detectable preclinical state followed by either (i) diagnosis of cancer due to symptoms or (ii) competing mortality, which refers to death from a competing risk [8]. The screen‐detectable preclinical cancer state is not a biological entity distinct from screening technology. Cancer biology is not sufficiently well understood to precisely define a preclinical cancer state [9].

Many cancer screening models allow missing detection of screen‐detectable preclinical cancer. However, this leads to the ill‐defined notion of missing screen‐detection in a state that is defined by screen‐detection. Nevertheless, some investigators postulate a screen‐detectable preclinical cancer state subject to technical errors [10], which I call a “true” screen‐detectable preclinical cancer (TPC) state. For example, the TPC state for breast cancer screening could be mammography without technical errors of poor imaging quality, poor position for imaging, or human error in reading images [10]. When postulating a TPC state, modelers specify a screening test sensitivity, which is the probability of screen‐detection given screening occurs in the presence a TPC state.

Two important quantities in cancer screening modeling are sojourn time and lead time. The sojourn time is the time in the screen‐detectable preclinical state if there were no screening and no competing mortality. The lead time is the time from screen‐detection to the development of symptomatic cancer if there were no screening and no competing mortality.

Many studies assume an exponential distribution for the sojourn time because it involves only one parameter, which is the reciprocal of the mean sojourn time (MST), computations are tractable, and the lead time follows an exponential distribution with the same parameter due to the memoryless property of the exponential distribution [11]. To allow more heterogeneity in the sojourn time distribution, some modelers specify two types of screen‐detectable preclinical states: progressive, which ends in a symptomatic cancer diagnosis and (ii) indolent, which remains to the maximum lifespan [12, 13, 14, 15]. These models typically assume an exponential distribution for the sojourn time in the progressive state and an additional parameter for the probability of being in the progressive state.

The challenge with modeling is to select reasonable assumptions such that all parameters can be precisely estimated with reasonable sample sizes. Even in a simple model with a TPC state and a parameter for screening test sensitivity, the estimated MST can be sensitive to a fixed screening test sensitivity [12] or can be imprecise due to a strong correlation with estimated screening test sensitivity [10].

## 3 CC Method

For estimating SOF from short‐term observational data on MCD tests, I introduce a new mathematical modeling approach, the cross‐sectional combination (CC) method. The name comes from using short‐term cross‐sectional screening data to estimate SOF for a yearly screening program. The CC method involves different assumptions from other mathematical modeling methods, which makes it a useful contribution.

### 3.1 Goal

To simplify the exposition, I discuss yearly screens for a particular age range, namely, 50 to 70. The maximum lifespan is set at 100 years. The goal is to use data from persons ages 50 to 70 who are screened at least twice, 1 year apart, to estimate the average SOF for yearly screening from ages 50 to 70.

### 3.2 Assumptions

The CC method requires the following assumptions.

Assumption 1 There exists in some persons an operational screen‐detectable preclinical cancer (OPC) state such that if the person is in the OPC state at a given age, the person will be in the OPC state at older ages if the person is alive and has not been diagnosed with symptomatic cancer.

Assumption 2 The probability of competing mortality is negligible before the oldest age in the yearly screening program, which is age 70 in the example.

Assumption 3 There is no screening with other modalities.

Assumption 4 Birth cohort does not affect screen detection rates.

Assumption 5 In the absence of competing mortality, the lead time in the OPC state follows an exponential distribution with parameter λ.

Assumptions 1 and 4 have been used with models for periodic screening evaluation [16].

Assumptions 1 and 2 contribute to graphical derivation of the formula for SOF. The idea behind Assumption 1 is that a positive test followed by a positive work‐up is a definitive state that would remain over time in the absence of screening. Before the start of the OPC state, there could a positive test and negative work‐up due to either a technical error or no preclinical cancer, which has no bearing on the analysis. Assumption 2 justifies ignoring overdiagnosis before age 70. Violation of Assumption 2 would lead to an underestimate of SOF. Assumptions 3 and 4 are needed in the SOF formula so that the probability of screen detection at a given age does not depend on calendar time. Assumption 5 is needed so that SOF formula is a function of a single parameter, simplifying the sensitivity analysis. I investigate violations of Assumption 5.

### 3.3 Graphical Depiction of OPC States

**FIGURE 1:**
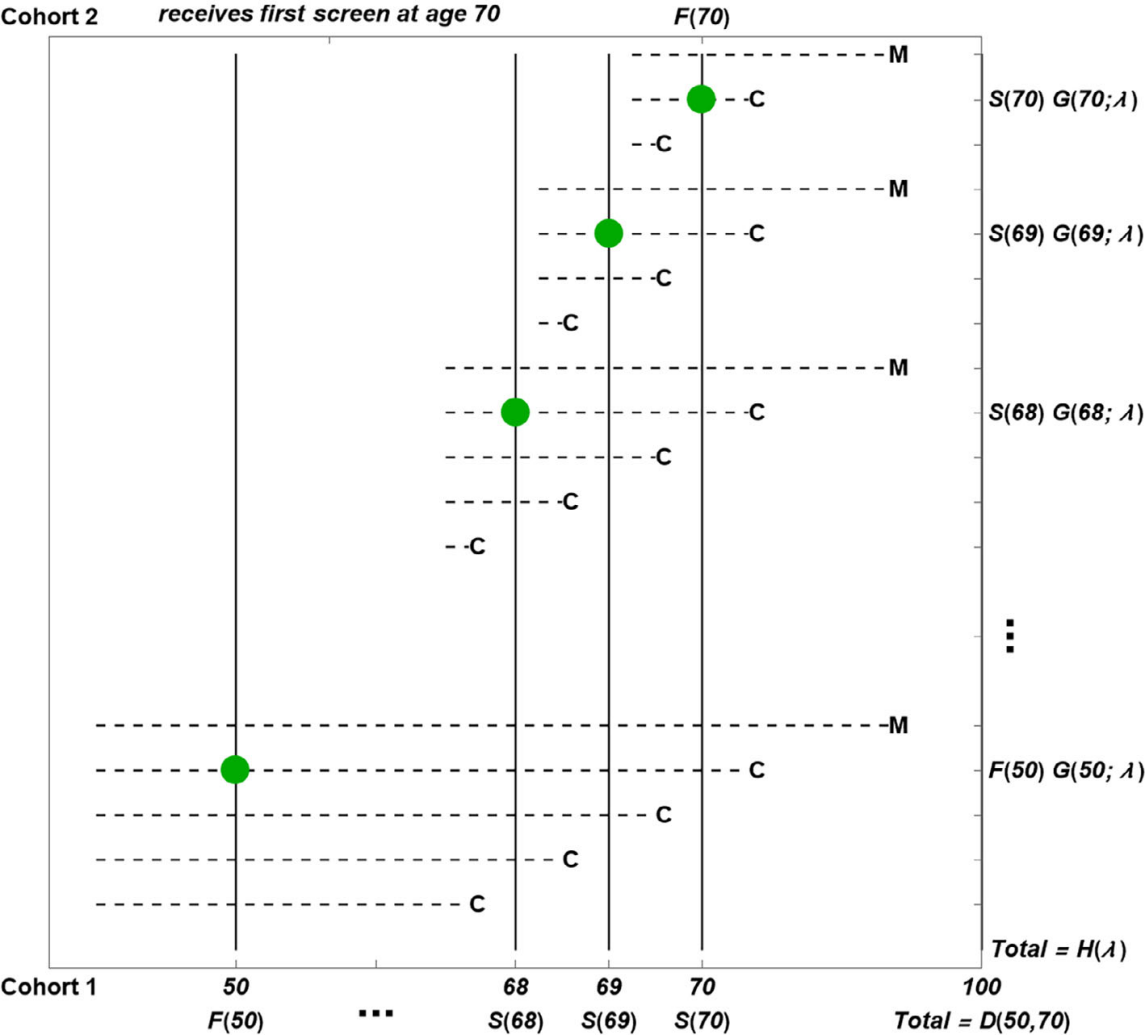
Derivation of *SOF*(λ) = {*F*(70) – *H*(λ)}/*D*(50, 70). Yearly screens occur from ages 50 to 70. The maximum lifespan is age 100. Horizontal dashed lines denote classes of OPC states that end in either (i) symptomatic cancer diagnosis in the presence of competing mortality, which is labeled C or (ii) competing mortality, which is labeled M. The large points denote the age at screen detection for an OPC state that ends in C in ages 70 to 100, and thus contributes to *H*(λ).

Figure 1 depicts classes of OPC states subject to censoring by competing mortality after age 70. Each horizontal dashed line corresponds to a class of OPC states starting and ending in a particular age interval for yearly screening from ages 50 to 70.

Assumption 1 implies the horizontal lines for OPC states are continuous and, if there is no screening, end in either (i) symptomatic cancer diagnosis in the presence of competing mortality, which is labeled C or (ii) competing mortality, which is labeled M. Assumption 2 implies that no OPC states end in M before age 70. Therefore, Figure 1 yields a key implication.

*Key implication*: The overdiagnosed OPC states for a screening program ending at age 70 are those OPC states that include age 70 and end in M.

The key implication fits the definition of overdiagnosis as detection of an OPC state on screening that would not have developed into symptomatic cancer in the absence of screening. Although there is no specification of progressive or indolent OPC states with the CC method, if progressive and indolent states were specified, the overdiagnosed OPC states would include (i) progressive OPC states that would have ended in symptomatic cancer diagnosis after M if there were no competing mortality and (ii) indolent OPC states that would remain to the maximum lifespan of age 100 if there were no competing mortality, with M appearing at age 100.

### 3.4 Age‐Specific Screen Detection Rates

The basic quantities for the CC method are age‐specific screen detection rates with a distinction between a first screen and a subsequent screen in a yearly screening program. Let

F(x)=probability of detectiononafirst screenatagex

S(x)=probability of detectiononasubsequent screenatagex

Based on Figure 1, screen detection on the first screen at age *x* occurs only in persons whose OPC states include age *x*, and screen detection on a subsequent screen at age *x*, occurs only in persons whose OPC state starts after age *x*–1 and includes age *x*. Consequently, one can also define *F*(*x*) and *S*(*x*) as

$$\begin{array}{cc} & F\left(x\right)=\text{probability}\;a\;\text{person}\;\mathrm{has}\;\text{an}\;\mathrm{OPC}\\ & \;\text{state that includes}\;\mathrm{age}\;x\; \end{array}$$

$$\begin{array}{cc} & S\left(x\right)=\text{probability}\;a\;\text{person}\;\mathrm{has}\;\text{an}\;\mathrm{OPC}\;\text{state that starts after}\\ & \;\mathrm{age}\;x-1\;\text{and includes}\;\mathrm{age}\;x\; \end{array}$$

### 3.5 SOF Formula

The formula for the average SOF for yearly screening from ages 50 to 70 involves two key quantities,

$$\begin{array}{cc} & F\left(70\right)=\text{probability of detection}\;\mathrm{on}\;a\;\\ & \;\text{first screen}\;\mathrm{at}\;\mathrm{age}\;70\; \end{array}$$

$$\begin{array}{ll} & \;=\text{probability}\;a\;\text{person}\;\mathrm{has}\;\text{an}\;\mathrm{OPC}\\ & \;\text{state that includes}\;\mathrm{age}\;70, \end{array}$$

$$\begin{array}{ll} & H\left(\lambda \right)=\text{probability}\;a\;\text{person}\;\mathrm{has}\;\text{an}\;\mathrm{OPC}\;\\ & \;\text{state that includes}\;\mathrm{age}\;70\;\text{and ends in}\;C \end{array}$$

Therefore *F*(70)–*H*(λ) is the probability a person has an OPC state that includes age 70 and ends in *M*, which, according to the key implication of Figure 1, is the probability of overdiagnosis for a screening program ending at age 70. The screening program ending at age 70 considered here is yearly screening from age 50 to 70. Let *D*(50, 70) = *F*(50) + ∑_*51*_^*70*^ *S*(*x*) denote the probability of screen detection among yearly screens from age 50 to age 70. Therefore, the average SOF for a program of yearly screens from ages 50 to 70 is

(1) SOF(λ)={F(70)−H(λ)}/D(50,70)

The next step is deriving a formula for *H*(λ) under Assumption 5. In this regard it is helpful to define *H*(λ) for yearly screening from age 50 to 70 in terms of counterfactual outcomes,

$$\begin{array}{ll} & H\left(\lambda \right)=\text{probability that}\;a\;\text{person screen}-\text{detected in}\\ & \;a\;\text{yearly screening program between}\;\mathrm{age}\;50\;\text{and}\;70\\ & \;\text{would},\text{in the absence of screening and in the presence}\\ & \;\text{of competing mortality after}\;\mathrm{age}\;70,\text{develop symptomatic}\\ & \;\text{cancer between ages}\;70\;\text{and}\;100. \end{array}$$

For example, one contribution to *H*(λ) would come from a person detected on a subsequent screen at age 60 who would have a lead time larger than 10 years and, in the presence of competing mortality after age 70, develop symptomatic cancer between age 70 and 100. Let *G*(*x*; λ) denote the probability that a person screen detected at age *x* would, in the absence of screening and the presence of competing mortality after age 70, develop symptomatic cancer between ages 70 and 100. As shown in Figure 1

(2) $$H\left(\lambda \right)=F\left(50\right)\;G\left(50;\lambda \right)+\sum_{x=51}^{70}S\left(x\right)\;G\left(x;\lambda \right)$$

Instead of specifying the distributions of starting ages for OPC states and sojourn times, which would be analogous to most cancer screening models, Equation (2) specifies probabilities of screen detection under yearly screening and a lead time distribution. Consequently, there is no need to specify a parameter for the distribution of starting ages for OPC states.

The final step is deriving a formula for *G*(*x*; λ). Let *Surv*(*u*) denote the probability surviving competing mortality to age *u* given survival to age 70. Let *y* denote the lead time, which, under Assumption 5, follows an exponential distribution. The probability the lead time, which is conditional on surviving competing mortality, ends in year *y* is *P*(*y*; λ) = exp(−λ *y*) – exp(−λ (*y* + 1)). Therefore,

(3) $$G\left(x;\lambda \right)=\sum_{y=70‐x}^{100‐x}\;\text{Surv}\left(x+y\right)\;P\left(y;\lambda \right)$$

Importantly, because the exponential distribution assumption only enters the formula for *SOF*(λ) via the term *H*(λ) in the numerator of Equation (1), misspecification of the exponential distribution would affect only *H*(λ) and not *F*(70), thereby limiting the impact of misspecification. In this sense, the estimate of *SOF*(λ) is robust to deviations from the exponential distribution assumption. Other mathematical models do not include a term for the probability of detection on a first screen at an older age, so do not share this robustness property.

### 3.6 SOF Estimation

I estimate SOF from cross‐sectional data from multiple birth cohorts whose ages at first screen range from age 50 to 70 and who receive at least 2 yearly screens. Preliminary estimates of *F*(*x*) and *S*(*x*) are the fraction of person screened at age *x* who were screen‐detected on first and subsequent screens, respectively. These estimates of *F*(*x*) and *S*(*x*) are incidence rates but, because screen‐detection is rare and competing mortality is negligible under Assumption 2, they approximate probabilities. Smoother and more precise estimates of *F*(*x*) and *S*(*x*) come from linear regressions of *F*(*x*) and *S*(*x*) on age *x*. Assumptions 3 and 4 justify applying these cross‐sectional estimates to a yearly screening program, where ages of screen‐detection are extrapolated to future calendar times. The estimate of *Surv*(*u*) comes from U.S. population data.

The Appendix A describes details on the estimation of *SOF*(λ) including the regression fit and computation of the 95% confidence interval. The SOF plot graphs estimated *SOF*(λ) and 95% confidence intervals versus MST, which equals 1/λ under the exponential distribution. If *SOF*(λ) is small for all reasonable values of MST, one would conclude that overdiagnosis is not a major concern.

### 3.7 Secondary Analysis

A secondary analysis uses the total number of interval cancers and total number of cancers detected on a subsequent screen to estimate an upper bound on MST. The secondary analysis assumes a population steady state for entering the OPC state. It also assumes that violations of the exponential distribution for the sojourn time in the OPC state have more probability mass after 1 year in the OPC state than with the exponential distribution. See the Supporting Information for details.

## 4 Results

Sufficient observational data from MCD tests are not yet available for applying the CC analysis. As proof‐of‐principle, I apply the CC method to lung cancer screening data and synthetic screening data.

### 4.1 Lung Cancer Screening

The first proof‐of‐principal analysis involves creating SOF plots using the following 4 data sets for lung cancer screening:

- *MLP x‐ray and cytology*, with counts from male heavy smokers ages 50 to 69 in the Mayo Lung Project (MLP) who received chest x‐ray and sputum cytology exams every 4 months for 6 years, summarized as yearly counts [17, 18, 19],
- *PLCO x‐ray*, with counts from participants ages 55 to 70 in Prostate Lung Colorectal, and Ovarian (PLCO) trial who received 3 yearly chest x‐rays [20],
- *NLST x‐ray*, with counts from participants ages 55 to 70 in the National Lung Screening Trial (NLST) who received 3 yearly chest x‐rays [21],
- *NLST CT*, with counts from participants ages 55 to 70 in the NLST who received 3 yearly computed tomography (CT) screens [21].

**FIGURE 2:**
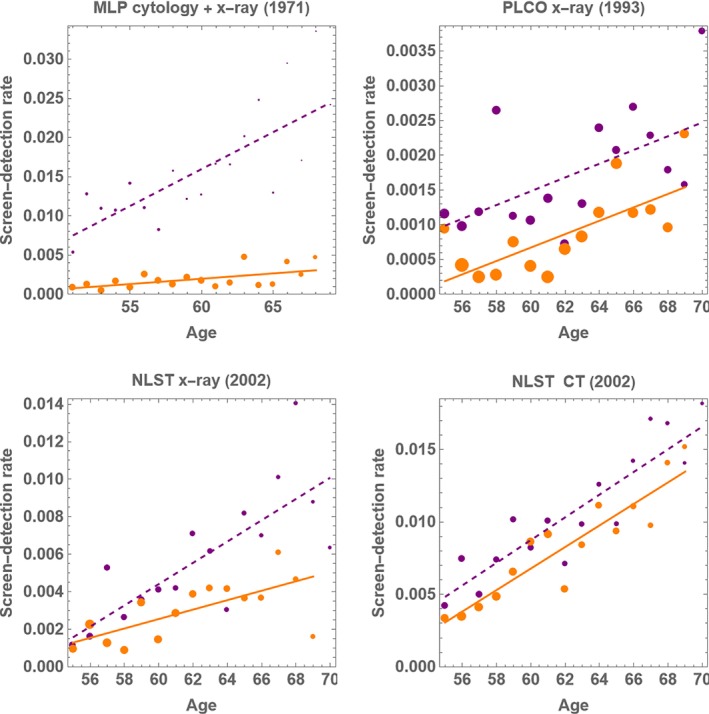
Linear regression plots for age‐specific screen detection with lung cancer screening data. Purple dashed line is first screen‐detection and orange solid line is subsequent‐screen detection. Point sizes are proportional to sample sizes.

**FIGURE 3:**
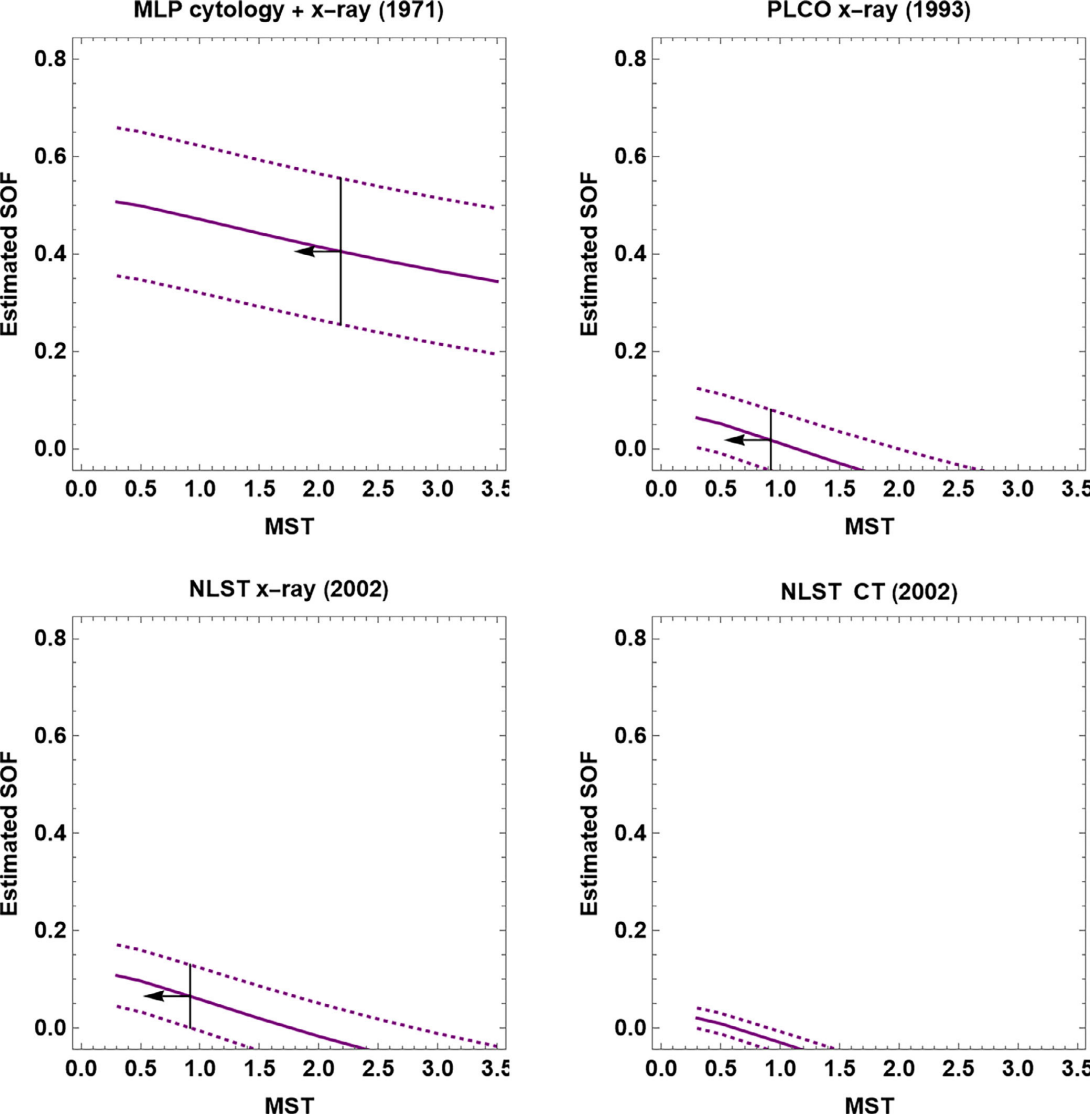
SOF plots of estimated SOF and 95% confidence interval (dashed lines) versus mean sojourn time (MST) in the OPC state for lung cancer screening data. The short‐dashed lines are 95% confidence intervals. The vertical lines with arrows pointing left denote MST upper bounds in the range of the plots.

Because these studies were conducted over 20 years ago, there was little lung cancer screening prior to the study, so Assumption 3 likely holds. Figure 2 shows regression lines fit to preliminary estimates of *F*(*x*) and *S*(*x*). In Figure 3, the SOF plots indicated small SOF with PLCO x‐ray, NLST x‐ray, and NLST CT, and moderate SOF with MLP x‐ray and cytology. Each plot also shows MST upper bounds as vertical lines with an arrow pointing left. The MST upper bounds were small except for NLST CT, which was outside the range of MST values.

### 4.2 Synthetic Screening Data

The second proof‐of‐principle analysis involves SOF plots for synthetic screening data. To create the synthetic data, I used a different model than assumed with the CC method, which simplified data generation and investigated the robustness of the method. For data generation I relaxed Assumption 5 and considered progressive and indolent OPC states. For the progressive state, I considered four distributions of sojourn time: Exponential (1), Exponential (1/2), Weibull (1.5, 1), and Weibull (1.5, 3). I considered two probabilities of indolent cancer: 0.1 and 0.4. For computational simplicity and to investigate robustness of the method, I specified the distribution of competing mortality as a triangular distribution with mode at 100. With the randomly generated OPC states, I superimposed 2 yearly screens starting at ages 50 to 70 and introduced competing mortality after age 70. This yielded synthetic data and a true average SOF denoted SOF_True_ for each distribution in the progressive state and each indolent state probability.

**FIGURE 4:**
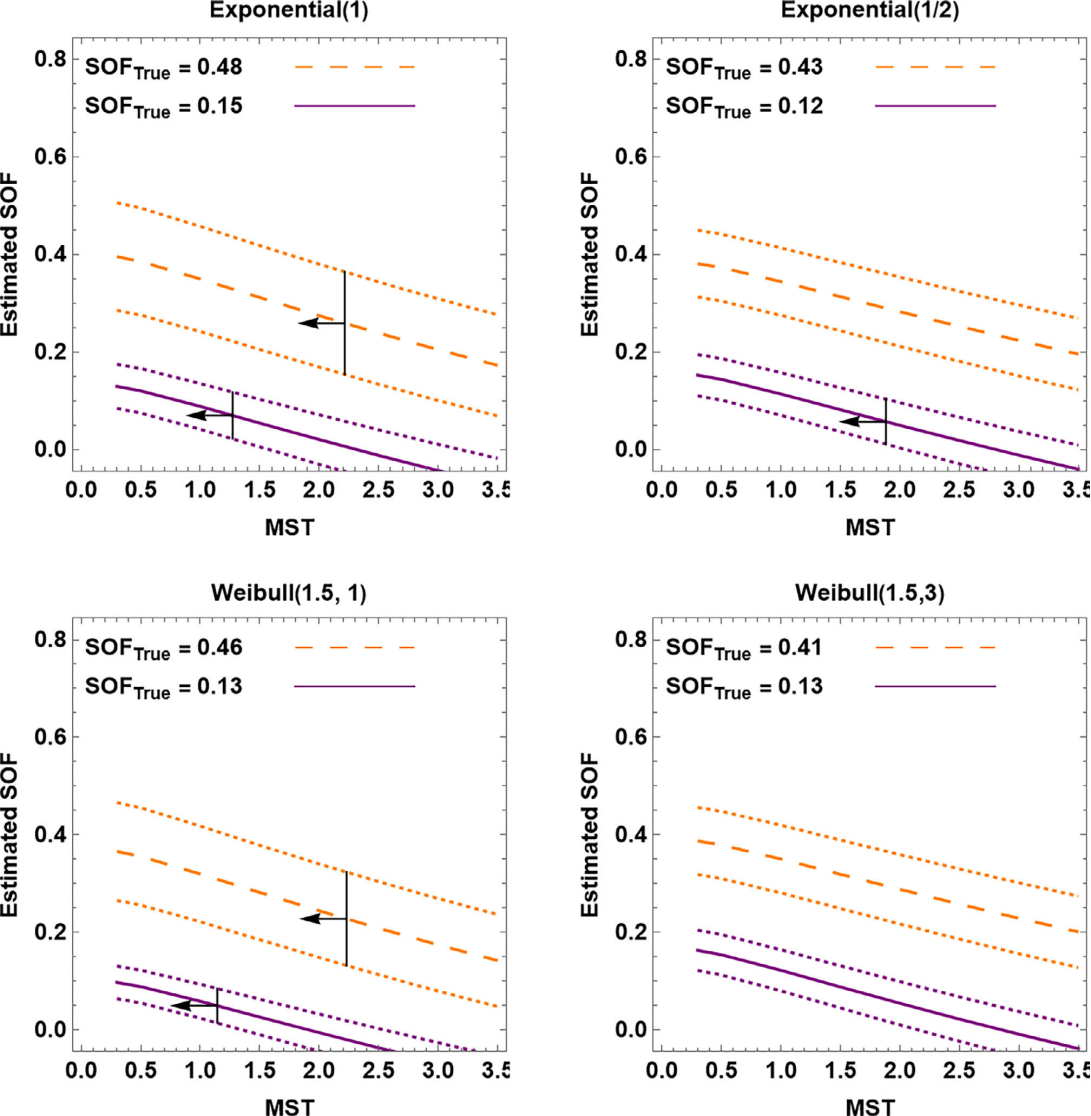
SOF plots of estimated SOF versus mean sojourn time (MST) in the OPC state for synthetic data. The short‐dashed lines are 95% confidence intervals. The vertical lines with arrows pointing left denote MST upper bounds in the range of the plots.

I computed SOF plots using the CC method (Figure 4). Despite the mismatch between the model for data generation and the model for the CC method, the SOF plots distinguished small from moderate SOF. Each plot shows MST upper bounds when the bound was within the range of the plot.

## 5 Discussion

Three types of evidence support the results of the SOF plot with lung cancer screening data. First, the similarity of the SOF plots for the 2 data sets with the same screening technology, namely x‐ray in PLCO and NSLT, is reassuring. Second, as discussed in the Supporting Information, the stop‐screen cumulative excess‐incidence estimate of SOF for MLP was 0.49 with 95% CI of (0.10, 0.87), which is consistent with moderate overdiagnosis indicated by the SOF plot. Third, estimates of MST for PLCO lung cancer screening based on a 5‐year follow‐up after the last screen were less than 1 year [22], in agreement with the MST upper bound from the secondary analysis.

There is no perfect method for quantifying overdiagnosis, and other modeling methods could usefully be applied to observational data from MCD tests. Each method requires different sets of assumptions or restrictions with different types of results. For example, other models can yield age‐specific SOF estimates [15] but may be less robust to deviations from the exponential distribution.

The CC method has 3 desirable attributes for estimating SOF with MCD tests. First, it can estimate average SOF using data from only 2 yearly screens at ages 50 to 70, making it possible to quickly obtain results from observational data from MCD tests. Second, the CC method uses age‐specific screen detection rates, which are easily obtained. Third, the CC method is robust to deviations from the assumed exponential distribution for sojourn time.

The CC method has 3 limitations. First it can only yield a range of SOF estimates over mean sojourn times rather than a precise estimate of SOF. This disadvantage is less serious for rapidly developing cancers, such as pancreatic cancer, where the MST is likely less than 1 or 2 years. The secondary analysis can also help to limit the range of MST. Second, to avoid violating Assumption 3, the CC method applies only to MCD tests for cancers for which there is no conventional screening. Third, the CC method assumes negligible competing mortality at ages younger than age 70.

In summary, the CC method is well suited to estimating SOF in MCD tests from only 2 yearly screens for cancers for which there is no conventional screening. Although the SOF plot showed a range of values of SOF rather than a single estimate, they could distinguish small from moderate SOF. As Welch and Black [23] noted, “even ‘best guess’ estimates about the magnitude of overdiagnosis play an important role in decision making.” With its unique formulation and assumptions, the CC method can complement other modeling approaches for quantifying overdiagnosis with MCD tests.

## Author Contributions

**Stuart G. Baker:** conceptualization; writing – original draft; writing – review and editing.

## Disclosure

Opinions expressed by the author are their own and this material should not be interpreted as representing the official viewpoint of the U.S. Department of Health and Human Services, the National Institutes of Health, the National Cancer Institute. The author thanks the National Cancer Institute for access to NCI's data collected by the Prostate, Lung, Colorectal and Ovarian Cancer Screening Trial and the National Lung Screening Trial. The statements contained herein are solely those of the authors and do not represent or imply concurrence or endorsement by NCI.

## Conflicts of Interest

The author declares no conflicts of interest.

## Supporting information


**Data S1** Supporting Information.

**Data S2** Supporting Information.

## Data Availability

The lung cancer screening data are available in the Supporting Information. The computer code, written in the Wolfram Programming Language, is available on request.

## Appendix A

### A.1

The data for SOF estimation are *d*_*F*_(*x*) = number of first‐screen detections at age *x*, *n*_*F*_(*x*) = number receiving the first screen at age *x*, *d*_*S*_(*x*) = number of subsequent‐screen detections at age *x*, *n*_*S*_(*x*) = number receiving a subsequent screen at age *x*. The preliminary estimates of *F*(x) and *S*(*x*) are $\hat{F}$(*x*) = *d*_*F*_(*x*)/*n*_*F*_(*x*) and $\hat{S}$(*x*) = *d*_*S*_(*x*)/*n*_*S*_(*x*). To smooth these estimates and increase precision, I fit *F*(*x*; *θ*) = *θ*_0_ + *θ*_1_ *x* with weights *n*_*F*_(*x*) to{*x*,$\;\hat{F}$(*x*)}, where *θ* = (*θ*_0_, *θ*_1_). I also fit *S*(*x*; *β*) = *β*_0_ + *β*_1_ *x* with weights *n*_*S*_(*x*) to {*x*, $\hat{S}$(*x*)}, where *β* = (*β*_0_, *β*_1_). Let $\hat{\theta }$ and $\hat{\beta }$ denote the regression estimates of *θ* and *β* with estimated covariance matrices $\hat{\mathrm{var}}$($\hat{\theta }$) and $\hat{\mathrm{var}}$($\hat{\beta }$), respectively. Let $\hat{H}\left(\lambda \right)$ denote the estimate of *H* (λ) based on *F*(*a*; $\hat{\theta }$), *S*(*x*; $\hat{\beta }$), and estimated *Surv*(*u*). The estimated SOF and its estimated variance are

(A1)
$$\hat{\mathrm{SOF}}\left(\lambda \right)=\left\{F\left(70;\hat{\theta }\right)–\hat{H}\left(\lambda \right)\right\}/\left\{F\left(50;\hat{\theta }\right)+\sum_{x=51}^{70}\;S\left(x;\hat{\beta }\right)\right\}$$

(A2)
$$\begin{array}{ll} \;\hat{\mathrm{var}}\left(\hat{\mathrm{SOF}}\left(\lambda \right)\right) & =\partial \hat{\mathrm{SOF}}\left(\lambda \right)/\partial \hat{\theta }\cdot \hat{\mathrm{var}}\left(\hat{\theta }\right)\cdot \partial \hat{\mathrm{SOF}}\left(\lambda \right)/\partial \hat{\theta }\\ & \;+\partial \hat{\mathrm{SOF}}\left(\lambda \right)/\partial \hat{\beta }\cdot \hat{\mathrm{var}}\left(\hat{\beta }\right)\cdot \partial \hat{\mathrm{SOF}}\left(\lambda \right)/\partial \hat{\beta } \end{array}$$

I symbolically computed the derivatives in Equation (A2). The 95% confidence interval for $\hat{\mathrm{SOF}}$(λ) is $\hat{\mathrm{SOF}}$ (λ) ± 1.96 $\hat{\mathrm{var}}$($\hat{\mathrm{SOF}}$(λ))^½^. An SOF plot graphs $\hat{\mathrm{SOF}}$(λ) and its 95% confidence interval versus 1/ λ.
