# Supplementary material for: Quantifying Overdiagnosis for Multicancer Detection Tests: A Novel Method
Source: Stat Med. 2024 Nov 26;43(30):5935–43. doi: 10.1002/sim.10285 (PMC11639630; doi:10.1002/sim.10285)
Supplement: Supplementary file 1 — Data S1 Supporting Information. [file SIM-43-5935-s002.docx]

August 29, 2024

**Quantifying overdiagnosis: A novel method for multicancer detection tests**

Supplementary materials

Outline

1. Secondary analysis
2. Generating synthetic data
3. Lung cancer screening data

4. Cumulative excess incidence estimates of SOF

1. **Secondary analysis**

For a secondary analysis, I use data from interval cancers to estimate the mean sojourn time in the progressive SDPC state. I consider a population steady-state scenario with *w* denoting the probability of entering OPC state at any calendar time *T*. The probability of an interval cancer is

*p_I_* (λ)= ∫*_T-1_^T^* *w* {1 – exp(–λ (*T* – *u*))} *du.*

Under the exponential distribution, the probability of screen detection on a subsequent screen is

*p_S_* (λ)= ∫*_T-1_^T^ w* exp{– λ (*T* – *u*)} *du*

To eliminate *w*, I consider the ratio *R*(λ)= *p_I_*(λ) / *p_S_*(λ). If there is more probability mass after 1 year in the OPC state than with the exponential distribution, the true ratio would have the form *R**(λ)= *p_I_*(λ) / (*p_S_*(λ) + *ε*), where *ε >*0. In Figure S1, I plot *R* and *R** with *ε =*0.2 versus MST = 1/ λ.


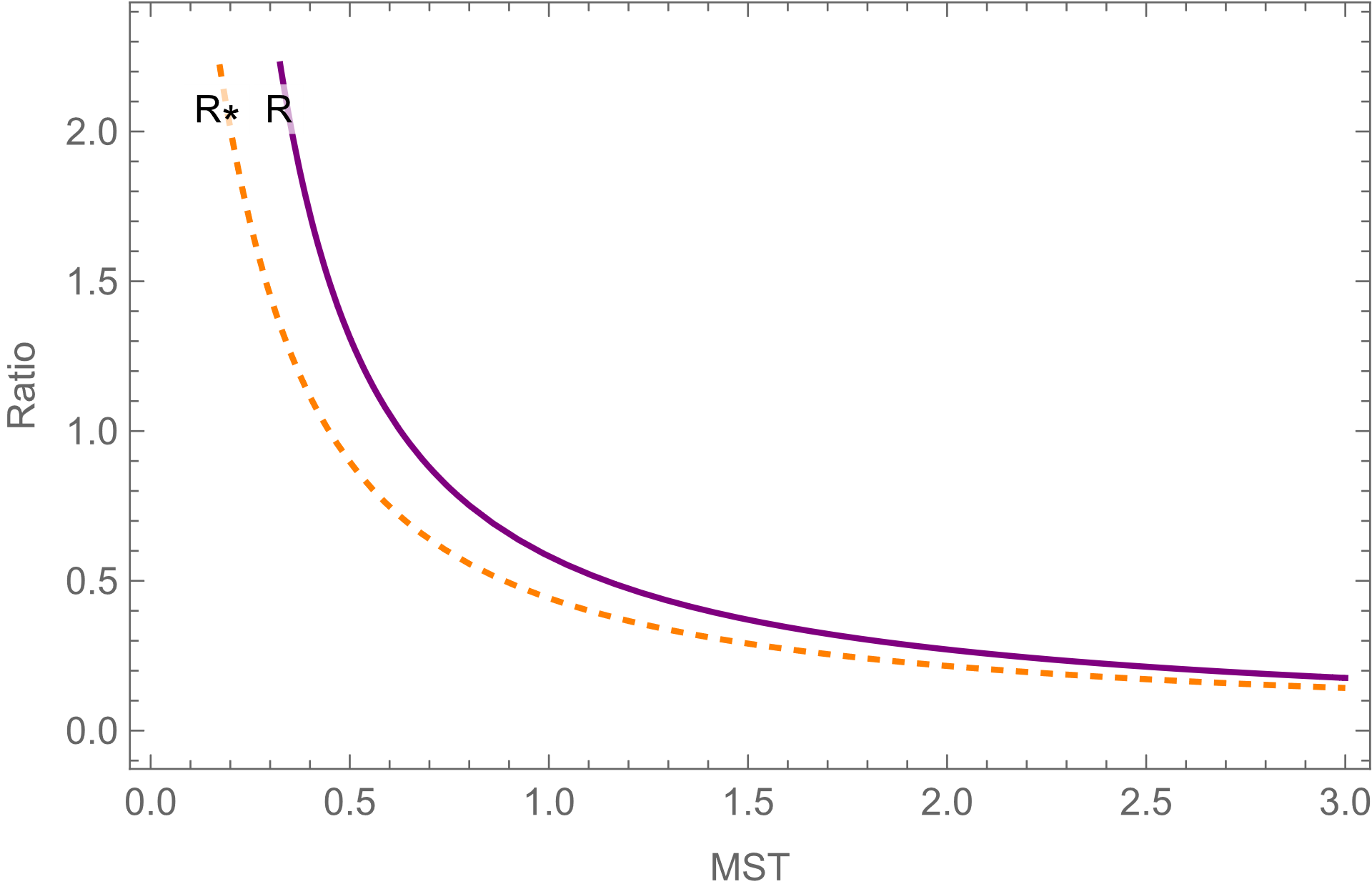


**Figure S1.** Plot of *R* and *R** versus MST

Based on Figure S1,  *R*=*R** requires a larger value of MST for *R* than *R**. Therefore, the MST computed based on *R* is an upper bound.

I define *M*(λ, SOF($\text{λ}$)) = *p_I_* / *p_S_,* which I estimate as $\hat{M}$ = (*d_I_* / *n_I_* ) / (*d_S_* / *n_S_*), where *d_I_* is the number of interval cancers, *n_I_* is the number screened at the start of the interval in which the interval cancer arises, *d_S_ = ∑_x_ d_S_*(*x*) is the number of subsequent-screen detected cancers, and *n_S_* = *∑_x_ n_S_*(*x*) is the number receiving the subsequent screen. To estimate λ, I use an EM algorithm with symbolic computing on the M-Step. For iteration *j*, which starts with *λ_j_*_,_ I compute *λ_j+1_* using

E-Step: Compute SOF(λ*_j_*)

M-Step: Solve $\hat{M}$ = *M*(λ, SOF(λ*_j_*)) for λ*_j+1_.*

**2. Synthetic data**

**2.1 Generation of synthetic data**

I generated the synthetic data using a progressive and indolent state model, which differs from the model used in the analysis. Let *Z* denote the age at entering the OPC state, which follows a triangular distribution from ages 45 to 70 with a mode at age 70. Let *G* =1 if the modified OPC state is indolent, with probability *p* and 0 if the SDPC state is progressive. I consider *p* = 0.10 or 0.40. Let *W* denote the sojourn time in the progressive SDPC state, which follows one of the 4 distributions discussed in the text (Figure S2). The random variable for the sojourn time in the OPC state is *Y* = *Z* +(1$\text{–}$*G*) W + *G* (100$\text{–}$Z). Let *C* denote the age of death from computing risk, which follows a triangular distribution from ages 45 to 100 with a mode at age 100. Let *z_i_*,  *y*_i_, and *c*_i_ denote elements of *Z*, *Y*, and *C* respectively.

From the distributions of *X* and *Y*, I generated *n* =200 pairs of starting and ending ages for SDPC states {*z_i_*, *y*_i_} where *i* indexes individual. Superimposing 2 screens 1 year apart at each age *x* from 50 to 70, I computed an indicator of first-screen detection, *d_Fi_*(*x*), an indicator of interval cancer, *d_Ii_*(*x*), and an indicator of subsequent-screen detection, *d_Si_*(*x*), as follows.

If *z_i_* < *x* and *y*_i_ ≥ *x* and *c*_i_ ≥ *x*, then *d_Fi_*(*x*) = 1 and 0 otherwise,

If *z_i_* > *x* and *y*_i_ < *x*+1 and *c*_i_ ≥ *x*+1, then *d_Ii_*(*x*) = 1 and 0 otherwise,

If *z_i_* > *x* and *z*_i_ < *x*+1 and *y*_i_ ≥ *x*+1 and *c*_i_ ≥ *x*+1, then *d_Si_*(*x*) = 1 and 0 otherwise.

Summing these indicators over all ages gives counts *d_F_*(*x*)=∑*_i_ d_Fi_*(*x*) and *d_S_*(*x*)=∑*_i_* *d_Si_*(*x*) that are used to estimate SOF. It also yields *d_I_* =∑*_x_* ∑*_i_ d_Ii_*(*x*), which is used in the secondary analysis.

I computed true SOF as the fraction of screen detections with *c*_i_ > *z_i_* and  *c*_i_ < *y*_i_.


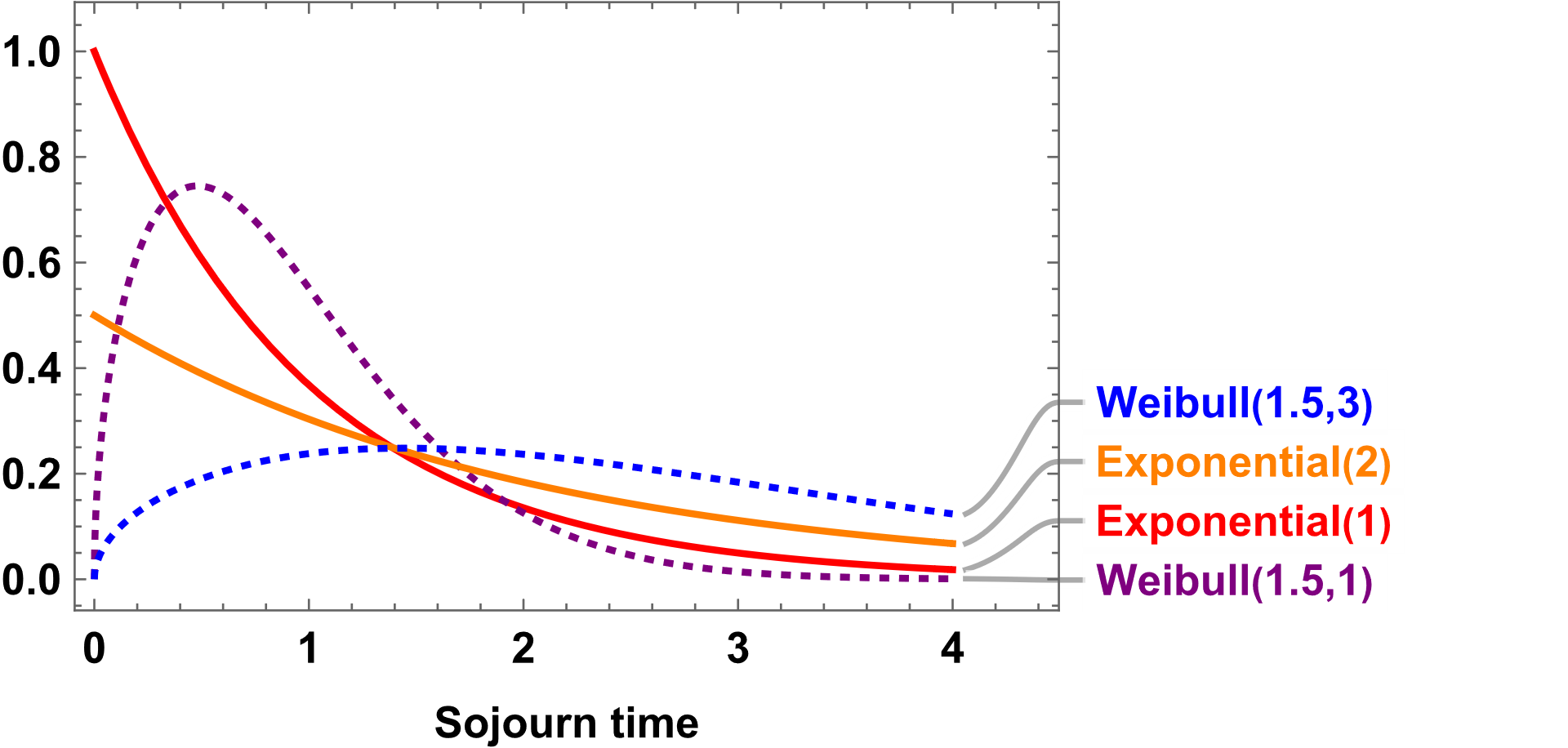


**Figure S2.** Distributions of sojourn time in progressive state for simulation

**3. Lung cancer screening data**

Tables S1 to S4 provide the lung cancer screening data.

**Table S1.** Age-specific cancer detection rates for x-ray and sputum cytology in MLP.

| Age | First screen | | Subsequent screen | |
| --- | --- | --- | --- | --- |
|  | Number positive | Number screened | Number positive | Number screened |
| 51 | 2 | 375 | 1 | 2363 |
| 52 | 5 | 392 | 2 | 2444 |
| 53 | 4 | 365 | 3 | 2414 |
| 54 | 4 | 374 | 1 | 2417 |
| 55 | 5 | 354 | 4 | 2419 |
| 56 | 4 | 364 | 2 | 2414 |
| 57 | 3 | 366 | 6 | 2417 |
| 58 | 5 | 317 | 4 | 2353 |
| 59 | 4 | 330 | 3 | 2385 |
| 60 | 4 | 315 | 5 | 2330 |
| 61 | 5 | 301 | 4 | 2275 |
| 62 | 5 | 302 | 2 | 2162 |
| 63 | 5 | 248 | 3 | 2072 |
| 64 | 6 | 242 | 9 | 1903 |
| 65 | 3 | 232 | 2 | 1768 |
| 66 | 5 | 170 | 2 | 1612 |
| 67 | 3 | 176 | 6 | 1446 |
| 68 | 5 | 149 | 3 | 1213 |
| 69 | 3 | 124 | 5 | 1068 |

**Table S2**. Age-specific screen detection data for x-ray screening in PLCO

| Age | First screen | | Subsequent screen | |
| --- | --- | --- | --- | --- |
|  | Number positive | Number screened | Number positive | Number screened |
| 55 | 6 | 5201 | 0 | 47 |
| 56 | 5 | 5142 | 5 | 5311 |
| 57 | 5 | 4244 | 4 | 9660 |
| 58 | 10 | 3789 | 2 | 8279 |
| 59 | 4 | 3573 | 2 | 7116 |
| 60 | 5 | 4731 | 5 | 6665 |
| 61 | 6 | 4369 | 3 | 7501 |
| 62 | 3 | 4185 | 2 | 8301 |
| 63 | 5 | 3836 | 5 | 7735 |
| 64 | 9 | 3759 | 6 | 7276 |
| 65 | 7 | 3384 | 8 | 6807 |
| 66 | 9 | 3346 | 12 | 6400 |
| 67 | 7 | 3070 | 7 | 5994 |
| 68 | 5 | 2804 | 7 | 5762 |
| 69 | 4 | 2546 | 5 | 5220 |
| 70 | 9 | 2378 | 11 | 4770 |

**Table S3.**  Age-specific screen detection data for x-ray screening in NLST

| Age | First screen | | Subsequent screen | |
| --- | --- | --- | --- | --- |
|  | Number positive | Number screened | Number positive | Number screened |
| 55 | 3 | 2633 | 1 | 814 |
| 56 | 4 | 2509 | 3 | 3178 |
| 57 | 11 | 2089 | 10 | 4433 |
| 58 | 5 | 1914 | 5 | 3964 |
| 59 | 7 | 1959 | 3 | 3512 |
| 60 | 8 | 1961 | 12 | 3518 |
| 61 | 7 | 1672 | 5 | 3472 |
| 62 | 11 | 1552 | 9 | 3174 |
| 63 | 9 | 1466 | 11 | 2850 |
| 64 | 4 | 1325 | 11 | 2635 |
| 65 | 10 | 1223 | 10 | 2419 |
| 66 | 7 | 1004 | 8 | 2195 |
| 67 | 9 | 893 | 7 | 1911 |
| 68 | 12 | 856 | 10 | 1641 |
| 69 | 6 | 685 | 7 | 1508 |
| 70 | 4 | 633 | 2 | 1252 |

**Table S4.** Age-specific screen detection data for CT screening in NLST

| Age | First screen | | Subsequent screen | |
| --- | --- | --- | --- | --- |
|  | Number positive | Number screened | Number positive | Number screened |
| 55 | 11 | 2627 | 0 | 871 |
| 56 | 19 | 2550 | 11 | 3273 |
| 57 | 11 | 2203 | 16 | 4599 |
| 58 | 14 | 1896 | 17 | 4151 |
| 59 | 20 | 1969 | 18 | 3736 |
| 60 | 16 | 1946 | 23 | 3527 |
| 61 | 18 | 1787 | 31 | 3601 |
| 62 | 11 | 1547 | 30 | 3291 |
| 63 | 14 | 1427 | 16 | 2985 |
| 64 | 17 | 1352 | 22 | 2622 |
| 65 | 12 | 1220 | 28 | 2517 |
| 66 | 14 | 988 | 21 | 2246 |
| 67 | 16 | 936 | 21 | 1897 |
| 68 | 14 | 833 | 17 | 1748 |
| 69 | 10 | 712 | 22 | 1563 |
| 70 | 12 | 661 | 20 | 1319 |

**4. Cumulative excess incidence estimates of SOF**.

This section details the calculation of the cumulative excess incidence estimates of SOF for the MLP and PLCO lung cancer screening data.

For MLP at 20 years after randomization there were 425 cancers in the screened arm and 360 in the control arm and 143 screen detection. Using the formula in Baker and Prorok (2021), the cumulative excess incidence estimate of SOF was 0.49 with 95% CI of (0.10, 0.87).

For PLCO lung cancer screening at 20 years after randomization, there were 1907 cancer in the screened arm, 1816 cancers in the control arm, and 310 screen detections. The cumulative excess incidence estimated SOF was 0.33 with 95% confidence interval (0.00, 0.75), which is too wide to be informative.
