# Supplementary material for: Quantifying Overdiagnosis for Multicancer Detection Tests: A Novel Method
Source: Stat Med. 2024 Nov 26;43(30):5935–43. doi: 10.1002/sim.10285 (PMC11639630; doi:10.1002/sim.10285)
Supplement: Supplementary file 2 — Data S2 Supporting Information. [file SIM-43-5935-s001.docx]

September 1, 2024

Quantifying overdiagnosis: A novel method for multicancer detection tests

Statistical software

Stuart G. Baker

Requirement: Mathematica Version 13 or later

To run the program:

copy all files overj*.m into some folder called "FOLDER"

start a new Mathematica session

type SetDirectory["FOLDER"]

type << overj.m

type OverJ[parsym,True]

Downloads

| 1 | overj.m | Calls other packagtes | Overj[parsym, newfitQ] |
| --- | --- | --- | --- |
| 2 | overjplotsof.m | SOF plot | OverPlotSOF[], |
| 3 | overjplotfs.m | Plots of regression lines F(x), S(x) | OverPlotFS[] |
| 4 | overjsof.m | Core computation | GenSOF[] |
| 5 | overjmst.m | Compute MST | OverJSimMST[], OverLungMST[] |
| 6 | overjsurv.m | Population survival data | GenSurv[] |
| 7 | overjdata.m | Calls data functions | GenData[] |
| 8 | overjsim.m | Generate simulated data | GenSimE[ ], GenSimW[] |
| 9 | overjdataSEI.m | Synthetic data, lung data | GenSEIDataSim…. etc |
| 10 | overjdataRCT.m | For Supplementary analysis | OverLungRCT[] |
| 11 | overjfig | Create synthetic data set | PlotFig1[], PlotFigS2,PlotFigS1 |

Disclaimer

This code is provided "as is", without warranty of any kind, express or implied, including but not limited to the warranties of merchantability, fitness for a particular purpose and non-infringement. In no event shall the NCI or the individual developers be liable for any claim, damages or other liability of any kind. Use of this code by recipient is at recipient's own risk. NCI makes no representations that the use of the code will not infringe any patent or proprietary rights of third parties.

PACKAGE 1

(*:Title:: main *)

(*:Summary:: overj *)

(*:References:: *)

(*Date 2024 Stuart G. Baker *)

BeginPackage["overj`", "overjdata`", "overjdataRCT`","overjfig`",

"overjplotsof`", "overjsof`", "overjsim`",

"overjdataSEI`","overjmst`", "overjsurv`",

"overjplotfs`"]

OverJ::usage ="OverJ[parsym,newfitQ]"

OverLung::usage ="OverLung[parsym,newfitQ]"

OverSim::usage ="OverLung[parsym,newfitQ]"

Clear[OverJ,OverLung,OverSim]

Begin["Private`"]

OverJ[parsym_,newfitQ_]:=

Module[{plotFig1,plotFig2,plotFig3,plotFig4},

(*Fig1*)

Print["Figure 1"];

plotFig1=PlotFig1[];

ExportPlot[plotFig1,"Fig1"];

(*Fig2*)

Print[" "];

Print["Figure 2"];

plotFig2=OverLungFS[];

ExportPlot[plotFig2,"Fig2"];

(*Fig3*)

Print[" "];

Print["Figure 3"];

plotFig3=OverLung[parsym,newfitQ];

ExportPlot[plotFig3,"Fig3"];

(*Fig4*)

Print[" "];

Print["Figure 4"];

plotFig4=OverSim[parsym,newfitQ];

ExportPlot[plotFig4,"Fig4"];

(*Supplement*)

Print[" "];

Print["Supplement"];

PlotFigS1[];

PlotFigS2[];

OverLungRCT[];

Return[Null]]

ExportPlot[plot_,name_]:=

Module[{namex},

Print[plot];

namex=StringJoin[name,".tif"];

Print["exporting ", namex];

Export[namex,plot];

Return[Null]]

OverLung[parsym_,newfitQ_:True]:=

Module[{adj,mstL,plotvec1,plotmatM1,plotM1},

adj=0;

SeedRandom[18];

show=False;

mstL=OverLungMST[parsym,newfitQ];

plotvec1=OverPlotSOF[1,parsym,10,20,mstL];

plotmatM1=Partition[plotvec1,2];

plotM1= Show[GraphicsGrid[plotmatM1],ImageSize->Large];

Return[plotM1]]

OverSim[parsym_,newfitQ_:True]:=

Module[{plotvec1,plotvec2,plotvec3,adj,modnum,mstE,mstW,

plotFS1,plotFS2,plotFS3,

plotE1A, plotE1B,plotE2A,plotE2B,

plotW1A, plotW1B,plotW2A,plotW2B,

plotmatM,plotmatS1,plotmatS2,plotM,plotS1,plotS2},

adj=0;

simtype=1;

show=False;

If[simtype==1, nsim=200; agenum=20];

If[simtype==2, nsim=5000; agenum=20];

Print["SImulation size= ",nsim, " agenum =", agenum];

{mstE,mstW}=OverSimMST[parsym,nsim,agenum,newfitQ];

Print["SOF PLOT"];

SeedRandom[18];

plotvec2=OverPlotSOF[2,parsym,nsim,agenum,mstE];

SeedRandom[18];

plotvec3=OverPlotSOF[3,parsym,nsim,agenum,mstW];

Print[" "];

{plotE1A, plotE1B,plotE2A,plotE2B}=plotvec2;

{plotW1A, plotW1B,plotW2A,plotW2B}=plotvec3;

plotmat={{Show[plotE1A, plotE1B], Show[plotE2A,plotE2B]},

{Show[plotW1A, plotW1B],Show[plotW2A,plotW2B]}};

plot= Show[GraphicsGrid[plotmat],ImageSize->Large];

Return[plot]]

OverSimFS[simtype_:1]:=

Module[{plotvec1,plotvec2,plotvec3,adj,modnum,mstE,mstW,

plotFS1,plotFS2,plotFS3,

plotE1A, plotE1B,plotE2A,plotE2B,

plotW1A, plotW1B,plotW2A,plotW2B,

plotmatM,plotmatS1,plotmatS2,plotM,plotS1,plotS2},

adj=0;

show=False;

If[simtype==1, nsim=200; agenum=20];

If[simtype==2, nsim=5000; agenum=20];

Print["SImulation size= ",nsim, " agenum =", agenum];

Print["REGRESSION PLOTS"];

Print["Exponential"];

OverESimFS[nsim,agenum];

Print["Weibull"];

OverWSimFS[nsim,agenum];

Return[Null]]

End[]

EndPackage[]

Print["OverJ[parsym,newfitQ]"];

parsym={t0,t1,b0,b1};

PACKAGE 2

(*:Title:: overj *)

(*:Summary:: SOF *)

(*:References:: *)

(*Date 2024 Stuart G. Baker *)

BeginPackage["overjplotsof`","overjsof`",

"overjdata`", "overjdataRCT`","overjdataSEI`","overjsurv`"]

OverPlotSOF::usage ="PlotSOF[datanum,parsym,SOFupp]"

Clear[OverPlotSOF]

Begin["Private`"]

OverPlotSOF[set_,parsym_,nsim_,agenum_,mstvec_]:=

Module[{range,plotvec,plotmatM,plotM},

modnum=1; (*linear model*)

adj=0; (*use all the data*)

SeedRandom[18];

range=Range[4]+ (set-1) 4;

pair=Transpose[{range,mstvec}];

plotvec=PlotSOF[set,#,parsym,modnum,adj,nsim,agenum]& /@pair;

Return[plotvec]]

PlotSOF[set_,{datanum_,mst_},parsym_,modnum_,adj_,nsim_,agenum_]:=

Module[{ymax,min0,max0,incr0,

dataSEI,name2x,

j,meanvec,mat,

SOFest,SOFlow,SOFupp,

SOFestX,SOFlowX,SOFuppX,

plotx,plotlist,

size,plotrange,topnamex,

nameD,ps,meanpdfs,sofs, name0,name1,text0,text1,y0,y1,

plotest,plotlow,plotupp},

ymax=.7;

ymax=.8;

min0=1/3;

max0=3.5;

incr0=.3;

{dataSEI,name2x}=GenData[datanum,adj,nsim,agenum];

(*COMPUTE SE of SOF with SOF model*)

meanvec={.3,.5,1,1.5,2,2.5,3,3.5};

hvec=1/meanvec;

matZ=GenSOF[{dataSEI,name2x,datanum},parsym,#,modnum]& /@ hvec;

{SOFestZ,SOFlowZ,SOFuppZ,seZ}=Transpose[matZ];

(*COMBINE ESTIME and SE*)

SOFlowB=SOFestZ-1.96 seZ;

SOFuppB=SOFestZ+1.96 seZ;

(*PLOT*)

SOFestX=Transpose[{meanvec,SOFestZ}];

SOFlowX=Transpose[{meanvec,SOFlowZ}];

SOFuppX=Transpose[{meanvec,SOFuppZ}];

plotest=ListPlot[SOFestX,Joined->True];

If[datanum==6 || datanum==8 || datanum==10 || datanum==12,

color1=Orange;

plotest=ListPlot[SOFestX,PlotStyle->{color1,Dashing[{.04,.04}]}, Joined->True];

plotlow=ListPlot[SOFlowX,PlotStyle->{color1,Dashing[{.01,.01}]}, Joined->True];

plotupp=ListPlot[SOFuppX,PlotStyle->{color1,Dashing[{.01,.01}]}, Joined->True],

(*else*)

color2=Purple;

plotest=ListPlot[SOFestX,PlotStyle->color2,Joined->True];

plotlow=ListPlot[SOFlowX,PlotStyle->{color2,Dashing[{.01,.01}]}, Joined->True];

plotupp=ListPlot[SOFuppX,PlotStyle->{color2,Dashing[{.01,.01}]}, Joined->True]];

(*COMBINE*)

size=12;

plotrange={{0,max0},{0,ymax}};

topnamex=GenDataNameSOFShort[set,datanum,nsim,agenum];

{SOFM,SOFMlow,SOFMupp,seM}= GenSOF[{dataSEI,name2x,datanum},parsym,1/mst,modnum];

vline=Graphics@{Line[{{mst,SOFMlow},{mst,SOFMupp}}]};

aline=Graphics@{Arrowheads[.05],Arrow[{{mst,SOFM},{mst-.4,SOFM}}]};

(*TEXT IN PLOT*)

If[set==1,plotlist={plotest,plotlow,plotupp,aline,vline}];

If[set==2 || set==3,

{nameD,p,meanpdf,sof}=name2x;

sofs=ToString[Round[sof,.01]];

meanpdfs=ToString[Round[meanpdf,.1]];

If[Mod[datanum,2]==0,

y0= .98 ymax;

sofx="\!\(\*SuperscriptBox[\(SOF\), \(*\)]\)";

mstx="\!\(\*SuperscriptBox[\(MST\), \(*\)]\)";

name0=Style[StringJoin[sofx," = ",sofs," ",mstx," = ",meanpdfs],Bold,size];

x00=2.4;

sofx="\!\(\*SubscriptBox[\(SOF\), \(True\)]\)";

mstx="\!\(\*SubscriptBox[\(MST\), \(True\)]\)";

name0=Style[StringJoin[sofx," = ",sofs],Bold,size];

x00=1.8;

line0=Graphics@{color1,Dashing[{.04,.04}],Line[{{x00,y0},{x00+1,y0}}]},

(*else*)

y0= .88 ymax;

sofx="\!\(\*SuperscriptBox[\(SOF\), \(*\)]\)";

mstx="\!\(\*SuperscriptBox[\(MST\), \(*\)]\)";

name0=Style[StringJoin[sofx," = ",sofs," ",mstx," = ",meanpdfs],Bold,size];

sofx="\!\(\*SubscriptBox[\(SOF\), \(True\)]\)";

mstx="\!\(\*SubscriptBox[\(MST\), \(True\)]\)";

name0=Style[StringJoin[sofx," = ",sofs],Bold,size];

x00=1.8;

line0=Graphics@{color2,Line[{{x00,y0},{x00+1,y0}}]}];

(*SOF MST label*)

text0=Graphics@Text[name0,{.03, y0},{-1,0}];

plotlist={plotest,plotlow,plotupp,text0,line0,aline,vline}];

(*FINAL PLOT*)

plotx=Show[plotlist,Frame->True,AspectRatio->1,Axes->None,

FrameLabel->{"MST","Estimated SOF",topnamex,None},

PlotRange->plotrange,LabelStyle->{Black,Bold,size}];

Return[plotx]]

End[]

EndPackage[]

PACKAGE 3

(*:Title:: overdiagsim *)

(*:Summary:: *)

(*:References:: *)

(*Date 2023 Stuart G. Baker *)

BeginPackage["overjplotfs`","overjdata`","overjdataRCT`","overjdataSEI`"]

OverLungFS::usage ="OverDiagSim[m,p]"

OverESimFS::usage ="OverDiagSim[m,p]"

OverWSimFS::usage ="OverDiagSim[m,p]"

Clear[OverLungFS,OverESimFS,OverWSimFS]

Begin["Private`"]

OverLungFS[]:=

Module[{adj,set,plotvec,range,plotmat,plotsetx,plotx,agenum},

adj=0;

set=1;

range=Range[4]+ (set-1) 4;

agenum=20;

plotvec=PlotFS[set,#,adj,nsim,agenum]& /@ range;

plotmat=Partition[plotvec,2];

plotsetx=GraphicsGrid[plotmat];

plotx=Show[plotsetx,ImageSize->Large];

Return[plotx]]

OverESimFS[nsim_,agenum_]:=

Module[{adj,set,plotvec,range,plotmat,plotsetx,plotx},

adj=0;

set=2;

range=Range[4]+ (set-1) 4;

plotvec=PlotFS[set,#,adj,nsim,agenum]& /@ range;

plotmat=Partition[plotvec,2];

plotsetx=GraphicsGrid[plotmat];

plotx=Show[plotsetx,ImageSize->Large];

Print[plotx];

Return[Null]]

OverWSimFS[nsim_,agenum_]:=

Module[{plotvec,range,plotmat,plotsetx,plotx},

adj=0;

set=3;

range=Range[4]+ (set-1) 4;

plotvec=PlotFS[set,#,adj,nsim,agenum]& /@ range;

plotmat=Partition[plotvec,2];

plotsetx=GraphicsGrid[plotmat];

plotx=Show[plotsetx,ImageSize->Large];

Print[plotx];

Return[Null]]

PlotFS[set_,datanum_,adj_,nsim_,agenum_]:=

Module[{dataSEI,dataRCT,gvec,matSEI,mat,plot,dataSEI0,u,r,

name,age0,Favg0,Savg0,Favg1,Savg1,nF,nS,nI,xS,xI, k,Fvec,Svec,nvecF,nvecS},

(*Data for model*)

{dataSEI,name2x}=GenData[datanum,adj,nsim,agenum];

{dataname,age0,Favg0,Savg0,Favg1,Savg1,nF,nS,nI,xS,xI, k,Fvec,Svec,nvecF,nvecS,survtype}=dataSEI;

manuQ=False;

sizeMS=8;

aicDIFB=0;

topname=GenDataNameSOF[set,datanum,nsim,agenum];

plotFS=PlotFSCore[age0,Fvec,Svec,nvecF,nvecS,manuQ,aicDIFB,topname,sizeMS];

Return[plotFS]]

PlotFSCore[age_,Fvec_,Svec_,nvecF_,nvecS_,manuQ_,aic_,dataname_,sizeMS_]:=

Module[{size,styleF,styleS,pairF,pairS,plotF,plotS,topname,

modF1,modF2,modS1,aicF1,aicF2,aicS1,

maxy,minage,maxage,lineF,lineS,x1,x2,y1,y2,key,plot},

size=13;

(*plot points*)

styleF=Purple;

styleS=Orange;

pairF=Transpose[{age,Fvec}];

pairS=Transpose[{age,Svec}];

age0=Drop[age,-1];

Svec0=Drop[Svec,1];

pairS0=Transpose[{age0,Svec0}];

nvecS0=Drop[nvecS,1];

triS=Transpose[{age0,Svec0,nvecS0}];

plotS=GenS[#,styleS]& /@ triS;

triF=Transpose[{age,Fvec,nvecF}];

plotF=GenF[#,styleF]& /@ triF;

(*plot fit*)

b=age[[-1]];

a=age[[1]];

dash=Dashing[{.02,.02}];

nvecF0=nvecF/Total[nvecF];

modF1=LinearModelFit[pairF,x,x, Weights->nvecF];

modF2=LinearModelFit[pairF,{x,x^2},x, Weights->nvecF];

plotF1=Plot[modF1[x], {x, a, b}, PlotStyle->{dash,styleF}];

plotF2=Plot[modF2[x], {x, a, b}, PlotStyle->styleF];

modS1=LinearModelFit[pairS0,x,x, Weights->nvecS0];

plotS1=Plot[modS1[x], {x, a, b-1}, PlotStyle->styleS];

aicF1=modF1["AIC"];

aicF2=modF2["AIC"];

aicS1=modS1["AIC"];

rF1=modF1["RSquared"];

rF2=modF2["RSquared"];

rS1=modS1["RSquared"];

topname=name;

maxy=Max[Fvec]//N;

minage=Min[age];

maxage=Max[age];

x1=minage+(maxage-minage) .02;

x2=minage+(maxage-minage) .4;

y1=.95 maxy;

y2=.85 maxy;

y3=.75 maxy;

aicF1s=ToString@Round[aicF1,.1];

aicF2s=ToString@Round[aicF2,.1];

aicS1s=ToString@Round[aicS1,.1];

rF1s=ToString@Round[rF1,.01];

rF2s=ToString@Round[rF2,.01];

rS1s=ToString@Round[rS1,.01];

name1=StringJoin["F linear: AIC=",aicF1s, " R2 = ",rF1s];

name2=StringJoin["F quad: AIC=",aicF2s, " R2 = ",rF2s];

name3=StringJoin["S linear: AIC=",aicS1s, " R2 = ",rS1s];

name1=StringJoin["F linear: AIC=",aicF1s];

name2=StringJoin["F quad: AIC=",aicF2s];

name3=StringJoin["S linear: AIC=",aicS1s];

text1=Graphics@{styleF,Text[Style[name1,size],{x1,y1},{-1,0}]};

text2=Graphics@{styleF,Text[Style[name2,size],{x1,y2},{-1,0}]};

text3=Graphics@{styleS,Text[Style[name3,size],{x1,y3},{-1,0}]};

plot=Show[plotF,plotS,plotF1,plotS1,AspectRatio->1,Frame->True,LabelStyle->{Bold,size},

FrameLabel->{"Age","Screen-detection rate",dataname,None}];

Return[plot]]

GenF[{x_,F_,n_},style_]:=Graphics@{PointSize[.0005 Sqrt[n]],style, Point[{x,F}]}

GenS[{x_,S_,n_},style_]:=Graphics@{PointSize[.0005 Sqrt[n]],style, Point[{x,S}]}

GenCircle[{x_,S_,n_}]:=Graphics@{Circle[{x,S},.001 Sqrt[n]], Green}

GenKey[x1_,x2_,y1_,y2_,y3_,styleF_,styleS_,manuQ_,aic_,size_,dataname_]:=

Module[{aics,style1,style2,text1,text2,text3, name1,name2,name3,line1,line2,line3,res},

(*not used*)

aics=ToString@Round[aic,.1];

name2=Style["First screen",size];

name3=Style["Subsequent screen",size];

namex2=StringJoin["AIC difference = ",aics];

name2=Style["First screen ",size];

name3=Style["Subsequent screen",size];

point1=Graphics@{styleF, PointSize[Large],Point[{x1,y1}]};

point2=Graphics@{styleS,Line[{{x1,y2},{x2,y2}}]};

(*revised*)

name1=Style[dataname,size];

text1=Graphics@{Black,Text[name1,{x1,y1},{-1,0}]};

text2=Graphics@{styleF,Text[name2,{x1,y2},{-1,0}]};

text3=Graphics@{styleS,Text[name3,{x1,y3},{-1,0}]};

line1=Graphics@{styleF, Line[{{x1,y1},{x2,y1}}]};

line2=Graphics@{styleF,Line[{{x1,y2},{x2,y2}}]};

line3=Graphics@{styleS,Line[{{x1,y3},{x2,y3}}]};

res={text2,text3};

Return[res]]

End[]

EndPackage[]

PACKAGE 4

(*:Title:: overj *)

(*:Summary:: overdiag *)

(*:References:: *)

(*Date 2024 Stuart G. Baker *)

BeginPackage["overjsof`","overjdata`", "overjdataRCT`","overjdataSEI`","overjsurv`"]

GenSOF::usage ="GenSOFlow[datanum,parsym,SOFupp]"

Clear[GenSOF]

Begin["Private`"]

GenSOF[{dataSEI_,name2x_,datanum_},parsym_,h0_,modnum_]:=

Module[{dataname,age0,Favg0,Savg0,Favg1,Savg1,nF,nS,nI,xS,xI, k,Fvec,Svec,nvecF,nvecS,survtype,

dataSEI0, a0,b0,c0, k0,

h, Q,Fmin,Fmax,Stot0,Fmin0,

pairF,modF1,parF1,varparF1,

pairS,modS1,parS1,varparS1,

tF0sym,tF1sym,tS0sym,tS1sym,

survM,survF,survB,hsym,

theta0,theta1,beta0,beta1,

rangeX,Sfit0,

Fmaxsym,Fminsym,SOFsym,dF0sym,dF1sym,dFvecsym,dSsym,

rule,dFvec0,dS0,Q0,

QsymZ,SsymZ,SOFsymZ,

se0,SOFlow0,SOFupps0},

(*Data for model*)

SetPrecision[$MinMachineNumber, $MachinePrecision]/2^20;

{dataname,age0,Favg0,Savg0,Favg1,Savg1,nF,nS,nI,xS,xI, kz,Fvec,Svec,nvecF,nvecS,survtype}=dataSEI;

dataSEI0={name,age0,Fvec,Svec,nvecF,nvecS,k};

(*Age range*)

a0=Min[age0];

b0=Max[age0];

c0=100;

k0=b0-a0;

(*FIT F MODEL*)

pairF=Transpose[{age0,Fvec}];

modF1=LinearModelFit[pairF,x,x, Weights->nvecF];

parF1=modF1["BestFitParameters"];

{theta0,theta1}=parF1;

varparF1=modF1["CovarianceMatrix"];

(*FIT S MODEL*)

(*For S(x) goes from a+1 to b*)

pairS=Transpose[{Drop[age0,1],Drop[Svec,1]}];

modS1=LinearModelFit[pairS,x,x, Weights->Drop[nvecS,1]];

parS1=modS1["BestFitParameters"];

{beta0,beta1}=parS1;

varparS1=modS1["CovarianceMatrix"];

(*RULE*)

{tF0sym,tF1sym,tS0sym,tS1sym}=parsym;

rule={ tF0sym->theta0, tF1sym->theta1, tS0sym->beta0, tS1sym->beta1};

(*F min and max*)

Fmin=modF1[a0];

Fmax=modF1[b0];

(*SOF VARIANCE*)

{xF0,xF1,xS0,xS1}=QFunctionSET[h0,{a0,b0,c0},{parF1,parS1},Fmin,h0,datanum];

Fmaxsym=tF0sym + tF1sym b0;

Fminsym=tF0sym + tF1sym a0;

QsymZ=xF0 tF0sym + xF1 tF1sym + xS0 tS0sym + xS1 tS1sym;

k0=b0-a0;

SsymZ= Fminsym + k0 tS0sym + Total[a0+Range[k0]] tS1sym;

SOFsymZ= (Fmaxsym- QsymZ) /(Fminsym + SsymZ);

dF0Z= D[SOFsymZ,tF0sym]/.rule;

dF1Z= D[SOFsymZ,tF1sym]/.rule;

dS0Z= D[SOFsymZ,tS0sym]/.rule;

dS1Z= D[SOFsymZ,tS1sym]/.rule;

dFvecZ={dF0Z,dF1Z};

dSvecZ={dS0Z,dS1Z};

varSOFZ=dFvecZ . varparF1 . dFvecZ + dSvecZ . varparS1 . dSvecZ//N;

seZ=Sqrt[varSOFZ];

(*Alternative SOF estimate yields same result*)

Q0K=xF0 theta0 + xF1 theta1 + xS0 beta0 + xS1 beta1;

den0= Fmin+ k0 beta0 + Total[a0+Range[k0]] beta1;

SOF0K= (Fmax - Q0K)/(den0-Q0K);

(*SOF ESTIMATE*)

Q0=QFunctionEST[h0,{a0,b0,c0},{Fmin,parS1},datanum];

rangeX=a0+Range[k0];

Sfit0=(beta0 +beta1 rangeX);

Stot0=Total[Sfit0];

SOF0= (Fmax- Q0) /((Fmin + Stot0));

SOFlow0=SOF0 - 1.96 seZ;

SOFupp0=SOF0+ 1.96 seZ;

Return[{SOF0,SOFlow0,SOFupp0,seZ}]]

(*-----------------------------------------------*)

QFunctionSET[h_,{a0_,b0_,c0_},{parF1_,parS1_},Fmin_,h0_,datanum_]:=

Module[{survM,survF,surv,

xF0,xF1,theta0,theta1,HF,x,

xS0,xS1,beta0,beta1,HS,q},

{survM,survF,surv}=GenSurv[];

xF0=CoreF0[a0,h0,{a0,b0,c0}, {surv,Fmin},datanum];

xF1=CoreF1[a0,h0,{a0,b0,c0}, {surv,Fmin},datanum];

xS0=Total@Table[CoreS0[x,h0,{a0,b0,c0},{surv,parS1},datanum],{x,a0+1,b0}];

xS1=Total@Table[CoreS1[x,h0,{a0,b0,c0},{surv,parS1},datanum],{x,a0+1,b0}];

Return[{xF0,xF1,xS0,xS1}]]

CoreF0[x_,h_,{a0_,b0_,c0_},{surv_,Fmin_},datanum_]:=

Module[{beta0,beta1,y,res},

(*

If[datanum<5,

res=Total@Table[ vz[h,y] (surv[[x+y]]/surv[[b0]]) ,{y,b0-x,c0-x}],

res=Total@Table[ vz[h,y] ,{y,b0-x,c0-x}]];

*)

res=Total@Table[ vz[h,y] (surv[[x+y]]/surv[[b0]]) ,{y,b0-x,c0-x}];

Return[res]]

CoreF1[x_,h_,{a0_,b0_,c0_},{surv_,Fmin_},datanum_]:=

Module[{beta0,beta1,y,res},

(*

If[datanum<5,

res=Total@Table[ vz[h,y] (surv[[x+y]]/surv[[b0]]) x ,{y,b0-x,c0-x}],

res=Total@Table[ vz[h,y] x ,{y,b0-x,c0-x}]];

*)

res=Total@Table[ vz[h,y] (surv[[x+y]]/surv[[b0]]) x ,{y,b0-x,c0-x}];

Return[res]]

CoreS0[x_,h_,{a0_,b0_,c0_},{surv_,parS1_},datanum_]:=

Module[{res,y},

(*

If[datanum<5,

res=Total@Table[ vz[h,y] (surv[[x+y]]/surv[[b0]]) , {y,b0-x,c0-x}],

res=Total@Table[ vz[h,y] , {y,b0-x,c0-x}]];

*)

res=Total@Table[ vz[h,y] (surv[[x+y]]/surv[[b0]]) , {y,b0-x,c0-x}];

Return[res]]

CoreS1[x_,h_,{a0_,b0_,c0_},{surv_,parS1_},datanum_]:=

Module[{res,y},

(*

If[datanum<5,

res=Total@Table[ vz[h,y] (surv[[x+y]]/surv[[b0]]) x , {y,b0-x,c0-x}],

res=Total@Table[ vz[h,y] x , {y,b0-x,c0-x}]];

*)

res=Total@Table[ vz[h,y] (surv[[x+y]]/surv[[b0]]) x , {y,b0-x,c0-x}];

Return[res]]

vz[h_,y_]:=CDF[ExponentialDistribution[h],y+1] -CDF[ExponentialDistribution[h],y]

(*------------------------------------------------------------*)

QFunctionEST[h_,{a0_,b0_,c0_},{Fmin_,parS1_},datanum_]:=

Module[{HF,HS,q,x,survM,survF,surv},

{survM,survF,surv}=GenSurv[];

HF=CoreFM[a0,h,{a0,b0,c0}, {surv,Fmin},datanum];

HS=Total@Table[CoreSM[x,h,{a0,b0,c0},{surv,parS1},datanum],{x,a0+1,b0}];

q=HF + HS;

Return[q]]

CoreFM[x_,h_,{a0_,b0_,c0_},{surv_,Fmin_},datanum_]:=

Module[{beta0,beta1,y,res},

If[datanum<5,

res=Total@Table[ vz[h,y] (surv[[x+y]]/surv[[b0]]) Fmin,{y,b0-x,c0-x}],

res=Total@Table[ vz[h,y] Fmin,{y,b0-x,c0-x}]];

Return[res]]

CoreSM[x_,h_,{a0_,b0_,c0_},{surv_,parS1_},datanum_]:=

Module[{beta0,beta1,y,res},

{beta0,beta1}=parS1;

If[datanum<5,

res=Total@Table[ vz[h,y] (surv[[x+y]]/surv[[b0]]) (beta0+ beta1 x),{y,b0-x,c0-x}],

(*else*)

res=Total@Table[ vz[h,y] (beta0+ beta1 x),{y,b0-x,c0-x}]];

Return[res]]

End[]

EndPackage[]

PACKAGE 5

(*:Title:: SOFXdiagsim *)

(*:Summary:: *)

(*:References:: *)

(*Date 2024 Stuart G. Baker *)

(*GenData[] from overjdata*)

(*GenDataName[] from overjdata*)

BeginPackage["overjmst`","overjsof`", "overjdata`","overjsim`"]

OverLungMST::usage ="OverMST[parsym]"

OverSimMST::usage ="OverMST[parsym]"

Clear[OverLungMST,OverSimMST]

Begin["Private`"]

OverLungMST[parsym_,newfitQ_]:=

Module[{modum,nsim,agenum,matL,mstL},

Print[" "];

Print["COMPUTE MST in LUNG Data"];

modnum=1;

nsim=100;

agenum=20;

matL=OverLambdaX[1,parsym,modnum,nsim,agenum,newfitQ];

mstL=TabMat[matL,1];

Return[mstL]]

OverSimMST[parsym_,nsim_,agenum_,newfitQ_]:=

Module[{modum,

matMST1,matMST2,matMST3,

colname,matMST},

Print[" "];

Print["COMPUTE MST in SIMULATION: nsim agenum ",{nsim,agenum}];

modnum=1;

matE=OverLambdaX[2,parsym,modnum,nsim,agenum,newfitQ];

matW=OverLambdaX[3,parsym,modnum,nsim,agenum,newfitQ];

mstE=TabMat[matE,2];

mstW=TabMat[matW,3];

Print[" "];

Return[{mstE,mstW}]]

OverLambdaX[set_,parsym_,modnum_,nsim_,agenum_,newfitQ_]:=

Module[{storedname,mst,res},

storedname=StringJoin["mstname",ToString[set],".m"];

If[newfitQ==True,

res=OverLambda[set,parsym,modnum,nsim,agenum];

Print[" exporting ",storedname];

Export[storedname,res]];

(*RETRIEVE OLD DATA SET*)

If[newfitQ==False,

res=Quiet@Drop[ReadList[storedname],1][[1]];

Print["importing ",storedname]];

Return[res]]

OverLambda[set_,parsym_,modnum_,nsim_,agenum_]:=

Module[{range, mat,mat1,dataname,matx},

SetPrecision[$MinMachineNumber, $MachinePrecision]/2^20;

range=Range[4]+ (set-1) 4;

mat=MST[set,#,parsym,modnum,nsim,agenum]& /@ range;

Return[mat]]

MST[set_,datanum_,parsym_,modnum_,nsim_,agenum_]:=

Module[{xI2,nI2,xS2,nS2,m2,res2,est,low,upp,

dataname,ps,mst,res},

{xI2,nI2,xS2,nS2,m2,res2,est,low,upp}=GenLambda[datanum,parsym,modnum,nsim,agenum];

{dataname,ps,mst}=GenDataNameMST[set,datanum,nsim,agenum];

res={dataname,ps,mst,xI2,nI2,xS2,nS2,m2,res2,est};

Return[res]]

GenLambda[datanum_,parsym_,modnum_,nsim_,agenum_]:=

Module[{adj,dataSEI,name2x,x,h1,h2,

meanvec,

dataname,age0,Favg0,Savg0,Favg1,Savg1,nF,nS,nI,xS,xI, k,Fvec,Svec,nvecF,ncSve,survtype,evec,

hI,hS,dS1,h,hL,hU,m0,h0},

(*BASIC PARAMETERS*)

adj=0;

{dataSEI,name2x}=GenData[datanum,adj,nsim,agenum]//N;

{dataname,age0,Favg0,Savg0,Favg1,Savg1,nF,nS,nI,xS,xI, k,Fvec,Svec,nvecF,nvecS,survtype}=dataSEI;

dataCORE={dataSEI,name2x,datanum};

(*ESTIMATES*)

hI=xI/nI//N;

hS=(xS/nS)//N;

m0=hI/hS;

h=parsym[[-1]];

r=hI/hS;

varhS=hS (1-hS)/nS;

varhI=hI (1-hI)/nI;

logm= Log[hI]-Log[hS];

varlogm= varhI/hI^2 + varhS/hS^2;

selogm=Sqrt[varlogm];

mlow=Exp[logm - 1.96 selogm];

mupp=Exp[logm + 1.96 selogm];

beta=1;

h0=1;

hest=Nest[EstimateLambdaZOne[dataCORE,parsym,h,{beta,#,m0},modnum]&, h0,5];

hL=Nest[EstimateLambdaZOne[dataCORE,parsym,h,{beta,#,mlow},modnum]&, h0,5];

hU=Nest[EstimateLambdaZOne[dataCORE,parsym,h,{beta,#,mupp},modnum]&, h0,5];

{mest, mlow,mupp} =ToString[#]& /@ Round[{1/hest,1/hU, 1/hL},.1];

mres=StringJoin[mest,"(", mlow, ",", mupp,")"];

meanvec =Round[{xI,nI, xS, nS}] ~Join~ {Round[m0,.01]} ~Join~ Round[{1/hest,1/hU, 1/hL},.1];

evec={1/hest,1/hU, 1/hL};

meanvec =Round[{xI,nI, xS, nS}] ~Join~ {Round[m0,.01], mres} ~Join~ evec;

Return[meanvec]]

EstimateLambdaZOne[dataCORE_,parsym_,h_,{beta_,h0_,m0_},modnum_]:=

Module[{h1,SOF0},

{SOF0,SOFlow,SOFupp,seZ}=GenSOF[dataCORE,parsym,h0,modnum];

h1=EstimateLambda[h,{beta,SOF0,m0}];

Return[h1]]

EstimateLambda[h_,{beta_,SOF_,m0_}]:=

Module[{dI,dS,msym,rule,h0,SOFX},

dI=Integrate[1 - Exp[-h(1-x)], {x, 0, 1}];

dS=Integrate[Exp[-h(1- x)], {x, 0, 1}];

SOFX= Max[0,SOF];

msym=dI /dS;

rule0=Quiet@FindRoot[msym==m0,{h,1}];

h0=h/.rule0;

Return[h0]]

TabMat[mat_,set_]:=

Module[{matx,dataname,ps,meanpdfs,xI2,nI2,xS2,nS2,hrat,res2,

mst,mat1,colname},

matx=Transpose[mat];

{dataname,ps,meanpdfs,xI2,nI2,xS2,nS2,hrat,res2,mst}=matx;

If[set==1,

mat1=Transpose[{dataname,hrat,res2}];

colname={"name","hI/hS", "MST (95% CI)"};

Print@TableForm[mat1,TableHeadings->{None,colname}]];

If[set>1,

mat1=Transpose[{dataname,ps,meanpdfs,hrat,res2}];

colname={"name","IndolentFrac","MST-true","hI/hS", "MST (95% CI)"};

Print@TableForm[mat1,TableHeadings->{None,colname}]];

Return[mst]]

End[]

EndPackage[]

PACKAGE 6

(*:Title:: overj *)

(*:Summary:: survival tables U.S. *)

(*:References:: *)

(*Date 2024 Stuart G. Baker *)

BeginPackage["overjsurv`"]

GenSurv::usage="GenSurv"

Clear[GenSurv]

Begin["Private`"]

GenSurv[]:=

Module[{},

mat=

{{0,0.006081,100000,76.22,0.005046,100000,81.28},

{1,0.000425,99392,75.69,0.000349,99495,80.69},

{2,0.000260,99350,74.72,0.000212,99461,79.72},

{3,0.000194,99324,73.74,0.000166,99440,78.73},

{4,0.000154,99305,72.76,0.000137,99423,77.75},

{5,0.000142,99289,71.77,0.000122,99409,76.76},

{6,0.000135,99275,70.78,0.000111,99397,75.77},

{7,0.000127,99262,69.79,0.000103,99386,74.77},

{8,0.000116,99249,68.79,0.000098,99376,73.78},

{9,0.000104,99238,67.80,0.000095,99366,72.79},

{10,0.000097,99227,66.81,0.000095,99357,71.80},

{11,0.000106,99218,65.82,0.000102,99348,70.80},

{12,0.000144,99207,64.82,0.000116,99337,69.81},

{13,0.000220,99193,63.83,0.000139,99326,68.82},

{14,0.000323,99171,62.85,0.000170,99312,67.83},

{15,0.000437,99139,61.87,0.000204,99295,66.84},

{16,0.000552,99096,60.89,0.000240,99275,65.85},

{17,0.000675,99041,59.93,0.000278,99251,64.87},

{18,0.000806,98974,58.97,0.000319,99224,63.89},

{19,0.000939,98894,58.01,0.000360,99192,62.91},

{20,0.001079,98802,57.07,0.000405,99156,61.93},

{21,0.001215,98695,56.13,0.000451,99116,60.95},

{22,0.001327,98575,55.20,0.000491,99071,59.98},

{23,0.001406,98444,54.27,0.000523,99023,59.01},

{24,0.001461,98306,53.35,0.000549,98971,58.04},

{25,0.001507,98162,52.42,0.000574,98917,57.07},

{26,0.001557,98014,51.50,0.000604,98860,56.11},

{27,0.001610,97862,50.58,0.000642,98800,55.14},

{28,0.001668,97704,49.66,0.000690,98737,54.17},

{29,0.001732,97541,48.74,0.000748,98669,53.21},

{30,0.001795,97372,47.83,0.000810,98595,52.25},

{31,0.001858,97198,46.91,0.000871,98515,51.29},

{32,0.001923,97017,46.00,0.000931,98429,50.34},

{33,0.001992,96830,45.09,0.000988,98337,49.38},

{34,0.002064,96638,44.18,0.001044,98240,48.43},

{35,0.002145,96438,43.27,0.001105,98138,47.48},

{36,0.002231,96231,42.36,0.001171,98029,46.53},

{37,0.002316,96017,41.45,0.001235,97914,45.59},

{38,0.002398,95794,40.55,0.001295,97793,44.64},

{39,0.002482,95564,39.64,0.001356,97667,43.70},

{40,0.002580,95327,38.74,0.001422,97534,42.76},

{41,0.002697,95081,37.84,0.001501,97396,41.82},

{42,0.002828,94825,36.94,0.001596,97249,40.88},

{43,0.002976,94557,36.04,0.001709,97094,39.95},

{44,0.003146,94275,35.15,0.001841,96928,39.01},

{45,0.003340,93979,34.26,0.001989,96750,38.08},

{46,0.003567,93665,33.37,0.002153,96557,37.16},

{47,0.003833,93331,32.49,0.002333,96350,36.24},

{48,0.004143,92973,31.61,0.002530,96125,35.32},

{49,0.004499,92588,30.74,0.002746,95882,34.41},

{50,0.004890,92171,29.88,0.002981,95618,33.50},

{51,0.005321,91720,29.02,0.003241,95333,32.60},

{52,0.005810,91232,28.18,0.003530,95024,31.71},

{53,0.006363,90702,27.34,0.003853,94689,30.82},

{54,0.006973,90125,26.51,0.004208,94324,29.93},

{55,0.007629,89497,25.69,0.004591,93927,29.06},

{56,0.008322,88814,24.89,0.004997,93496,28.19},

{57,0.009049,88075,24.09,0.005426,93029,27.33},

{58,0.009806,87278,23.31,0.005876,92524,26.48},

{59,0.010595,86422,22.53,0.006348,91980,25.63},

{60,0.011452,85506,21.77,0.006883,91396,24.79},

{61,0.012358,84527,21.01,0.007457,90767,23.96},

{62,0.013255,83482,20.27,0.008010,90090,23.14},

{63,0.014126,82376,19.54,0.008520,89369,22.32},

{64,0.015006,81212,18.81,0.009031,88607,21.51},

{65,0.016001,79994,18.09,0.009617,87807,20.70},

{66,0.017124,78714,17.37,0.010328,86963,19.89},

{67,0.018298,77366,16.67,0.011167,86065,19.10},

{68,0.019519,75950,15.97,0.012158,85103,18.31},

{69,0.020847,74468,15.28,0.013312,84069,17.52},

{70,0.022381,72915,14.59,0.014673,82950,16.75},

{71,0.024185,71283,13.91,0.016221,81733,16.00},

{72,0.026266,69559,13.25,0.017905,80407,15.25},

{73,0.028660,67732,12.59,0.019714,78967,14.52},

{74,0.031401,65791,11.95,0.021714,77410,13.80},

{75,0.034618,63725,11.32,0.024080,75729,13.10},

{76,0.038263,61519,10.71,0.026831,73906,12.41},

{77,0.042190,59165,10.11,0.029855,71923,11.74},

{78,0.046367,56669,9.54,0.033151,69776,11.08},

{79,0.050948,54041,8.97,0.036829,67463,10.45},

{80,0.056237,51288,8.43,0.041122,64978,9.83},

{81,0.062360,48404,7.90,0.046102,62306,9.23},

{82,0.069226,45385,7.39,0.051683,59434,8.65},

{83,0.076884,42243,6.91,0.057896,56362,8.09},

{84,0.085452,38996,6.44,0.064863,53099,7.56},

{85,0.095062,35663,6.00,0.072731,49655,7.05},

{86,0.105829,32273,5.57,0.081626,46043,6.56},

{87,0.117838,28858,5.17,0.091644,42285,6.10},

{88,0.131138,25457,4.80,0.102840,38410,5.67},

{89,0.145751,22119,4.45,0.115236,34460,5.26},

{90,0.161678,18895,4.12,0.128837,30489,4.88},

{91,0.178905,15840,3.82,0.143633,26561,4.52},

{92,0.197408,13006,3.54,0.159606,22746,4.20},

{93,0.217149,10439,3.29,0.176731,19115,3.90},

{94,0.238080,8172,3.06,0.194973,15737,3.63},

{95,0.258821,6226,2.86,0.213413,12669,3.39},

{96,0.278966,4615,2.69,0.231752,9965,3.17},

{97,0.298092,3327,2.54,0.249663,7656,2.98},

{98,0.315762,2336,2.40,0.266801,5744,2.81},

{99,0.331550,1598,2.28,0.282809,4212,2.65},

{100,0.348128,1068,2.16,0.299778,3021,2.49},

{101,0.365534,696,2.05,0.317765,2115,2.34},

{102,0.383811,442,1.94,0.336830,1443,2.20},

{103,0.403001,272,1.83,0.357040,957,2.07},

{104,0.423151,163,1.73,0.378463,615,1.94},

{105,0.444309,94,1.63,0.401170,382,1.82},

{106,0.466524,52,1.54,0.425241,229,1.70},

{107,0.489851,28,1.45,0.450755,132,1.59},

{108,0.514343,14,1.37,0.477800,72,1.48},

{109,0.540060,7,1.28,0.506468,38,1.38},

{110,0.567063,3,1.21,0.536857,19,1.28},

{111,0.595417,1,1.13,0.569068,9,1.19},

{112,0.625187,1,1.06,0.603212,4,1.10},

{113,0.656447,0,0.99,0.639405,1,1.02},

{114,0.689269,0,0.92,0.677769,1,0.94},

{115,0.723732,0,0.86,0.718435,0,0.87},

{116,0.759919,0,0.80,0.759919,0,0.80},

{117,0.797915,0,0.74,0.797915,0,0.74},

{118,0.837811,0,0.68,0.837811,0,0.68},

{119,0.879701,0,0.63,0.879701,0,0.63}};

vec=Dimensions[#]& /@ mat;

{age,hM,nM,lifeM,hF,nF,lifeF}=Transpose[mat];

h=(hM+hF)/2;

surv=FoldList[Times, (1-h)];

survM=FoldList[Times, (1-hM)];

survF=FoldList[Times, (1-hF)];

Return[{survM,survF,surv}]]

End[]

EndPackage[]

Package 7

(*:Title:: overj *)

(*:Summary:: overdiag data *)

(*:References:: *)

(*Date 2024 Stuart G. Baker *)

BeginPackage["overjdata`","overjdataRCT`","overjdataSEI`","overjsim`"]

GenData::usage ="GenData[datanum]"

GenDataNameSOF::usage ="GenData[datanum]"

GenDataNameMST::usage ="GenData[datanum]"

GenDataNameX::usage ="GenData[datanum]"

GenDataNameSOFShort::usage="GenDataNameSOFShort"

Clear[GenDataNameSOF,GenNameMST,GenData,GenDataNameX,GenDataNameSOFShort]

Begin["Private`"]

GenData[datanum_,adj_,nsim_,agenum_]:=

Module[{dataSYN,dataRCT,gvec,matSYN,matRCT,mat,plot,dataSYN0,u,mean,n, show,

name,age,Favg0,Savg0,Favg1,Savg1,nF,nS,nI,xS,xI, k,Fvec,Svec,nvecF,nvecS,p0,p1},

If[datanum==1,

dataSYN=GenSEIDataMayo[adj,False];

name2x="Randomized excess incidence"];

If[datanum==2,

dataSYN=GenSEIDataPLCOL[adj,False];

name2x="Randomized excess incidence"];

If[datanum==3,

dataSYN=GenSEIDataNLSTX[adj,False];

name2x="Randomized excess incidence CT vs X"];

If[datanum==4,

dataSYN=GenSEIDataNLSTC[adj,False];

name2x="Randomized excess incidence CT vs X"];

p0=.1;

p1=.4;

agevec=Range[agenum]+50;

If[datanum==5,

{dataSYN,p,meanpdf,sof}=GenSEIDataSimE[adj,p0,1,nsim,False,agevec];

name2x={"Exponential",p,meanpdf,sof}];

If[datanum==6,

{dataSYN,p,meanpdf,sof}=GenSEIDataSimE[adj,p1,1,nsim,False,agevec];

name2x={"Exponential",p,meanpdf,sof}] ;

If[datanum==7,

{dataSYN,p,meanpdf,sof}=GenSEIDataSimE[adj,p0,2,nsim,False,agevec];

name2x={"Exponential",p,meanpdf,sof} ];

If[datanum==8,

{dataSYN,p,meanpdf,sof}=GenSEIDataSimE[adj,p1,2,nsim,False,agevec];

name2x={"Exponential",p,meanpdf,sof}] ;

If[datanum==9,

{dataSYN,p,meanpdf,sof}=GenSEIDataSimW[adj,p0,1,nsim,False,agevec];

name2x={"Weibull",p,meanpdf,sof}] ;

If[datanum==10,

{dataSYN,p,meanpdf,sof}=GenSEIDataSimW[adj,p1,1,nsim,False,agevec];

name2x={"Weibull",p,meanpdf,sof}] ;

If[datanum==11,

{dataSYN,p,meanpdf,sof}=GenSEIDataSimW[adj,p0,3,nsim,False,agevec];

name2x={"Weibull",p,meanpdf,sof}] ;

If[datanum==12,

{dataSYN,p,meanpdf,sof}=GenSEIDataSimW[adj,p1,3,nsim,False,agevec];

name2x={"Weibull",p,meanpdf,sof} ];

(*Here adj is mean*)

meanx=adj;

If[datanum==13,

{dataSYN,p,meanpdf,sof}=GenSEIDataSimE[0,p0,meanx,nsim,False,agevec];

name2x={"Exponential",p,meanpdf,sof} ];

If[datanum==14,

{dataSYN,p,meanpdf,sof}=GenSEIDataSimW[0,p0,meanx,nsim,False,agevec];

name2x={"Weibull",p,meanpdf,sof} ];

If[datanum==15,

{dataSYN,p,meanpdf,sof}=GenSEIDataSimE[0,p1,meanx,nsim,False,agevec];

name2x={"Exponential",p,meanpdf,sof} ];

If[datanum==16,

{dataSYN,p,meanpdf,sof}=GenSEIDataSimW[0,p1,meanx,nsim,False,agevec];

name2x={"Weibull",p,meanpdf,sof} ];

Return[{dataSYN,name2x}]]

GenDataNameSOF[set_,datanum_,nsim_,agenum_]:=

Module[{manuQ,showRCT,dataSEI,matRCT,name2x,topname,nameD,ps,msts,sofs,name},

adj=0;

{dataSEI,name2x}=GenData[datanum,adj,nsim,agenum];

dataname=dataSEI[[1]];

If[set==1, topname=dataname];

If[set==2 || set==3,{nameD,p,meanpdf,sof}=name2x;

ps=ToString[p];

sofs=ToString[Round[sof,.01]];

meanpdfs=ToString[Round[meanpdf,.1]];

name=StringJoin[" with PI = ",ps]];

If[set==2 && datanum==5, topname=StringJoin["Exponential(1)",name]];

If[set==2 && datanum==6, topname=StringJoin["Exponential(1)",name]];

If[set==2 && datanum==7, topname=StringJoin["Exponential(1/2)",name]];

If[set==2 && datanum==8, topname=StringJoin["Exponential(1/2)",name]];

If[set==3 && datanum==9, topname=StringJoin["Weibull(1.5, 1)",name]];

If[set==3 && datanum==10, topname=StringJoin["Weibull(1.5,1)",name]];

If[set==3 && datanum==11, topname=StringJoin["Weibull(1.5,3)",name]];

If[set==3 && datanum==12, topname=StringJoin["Weibull(1.5,3)",name]];

Return[topname]]

GenDataNameMST[set_,datanum_,nsim_,agenum_]:=

Module[{manuQ,showRCT,dataSEI,matRCT,name2x,topname,nameD,ps,msts,sofs},

adj=0;{dataSEI,name2x}=GenData[datanum,adj,nsim,agenum];

dataname=dataSEI[[1]];

If[set==1, topname=dataname; ps=" "; msts=" "];

If[set==2 || set==3,{nameD,p,meanpdf,sof}=name2x;

ps=ToString[p];

sofs=ToString[Round[sof,.1]];

msts=ToString[Round[meanpdf,.1]];

name=StringJoin[" with PI = ",sofs];

name=StringJoin[" with MST = ",ps]];

If[set==2 && datanum==5, topname="Exponential(1)"];

If[set==2 && datanum==6, topname="Exponential(1)"];

If[set==2 && datanum==7, topname="Exponential(1/2)"];

If[set==2 && datanum==8, topname="Exponential(1/2)"];

If[set==3 && datanum==9, topname="Weibull(1.5, 1)"];

If[set==3 && datanum==10, topname="Weibull(1.5,1)"];

If[set==3 && datanum==11, topname="Weibull(1.5,3)"];

If[set==3 && datanum==12, topname="Weibull(1.5,3)"];

Return[{topname,ps,msts}]]

GenDataNameSOFShort[set_,datanum_,nsim_,agenum_]:=

Module[{manuQ,showRCT,dataSEI,matRCT,name2x,topname,nameD,ps,msts,sofs,name},

adj=0;

{dataSEI,name2x}=GenData[datanum,adj,nsim,agenum];

dataname=dataSEI[[1]];

If[set==1, topname=dataname];

If[set==2 || set==3,{nameD,p,meanpdf,sof}=name2x;

ps=ToString[p];

sofs=ToString[Round[sof,.01]];

meanpdfs=ToString[Round[meanpdf,.1]];

name=StringJoin[" with SOF = ",sofs];

name=StringJoin[" with OP = ",ps]];

If[set==2 && datanum==5, topname=StringJoin["Exponential(1)"]];

If[set==2 && datanum==6, topname=StringJoin["Exponential(1)"]];

If[set==2 && datanum==7, topname=StringJoin["Exponential(1/2)"]];

If[set==2 && datanum==8, topname=StringJoin["Exponential(1/2)"]];

If[set==3 && datanum==9, topname=StringJoin["Weibull(1.5, 1)"]];

If[set==3 && datanum==10, topname=StringJoin["Weibull(1.5,1)"]];

If[set==3 && datanum==11, topname=StringJoin["Weibull(1.5,3)"]];

If[set==3 && datanum==12, topname=StringJoin["Weibull(1.5,3)"]];

Return[topname]]

GenDataNameX[set_,datanum_,nsim_,agenum_]:=

Module[{manuQ,showRCT,dataSEI,matRCT,name2x,topname,nameD,ps,msts,sofs,name},

adj=0;

{dataSEI,name2x}=GenData[datanum,adj,nsim,agenum];

dataname=dataSEI[[1]];

If[set==1, topname=dataname];

If[set==2 || set==3,{nameD,p,meanpdf,sof}=name2x;

ps=ToString[p];

sofs=ToString[Round[sof,.01]];

meanpdfs=ToString[Round[meanpdf,.1]];

name=StringJoin[" with SOF = ",sofs];

name=StringJoin[" with OP = ",ps]];

If[set==2 && datanum==5, topname=StringJoin["Synthetic data 1"]];

If[set==2 && datanum==6, topname=StringJoin["Synthetic data 1"]];

If[set==2 && datanum==7, topname=StringJoin["Synthetic data 2"]];

If[set==2 && datanum==8, topname=StringJoin["Synthetic data 2"]];

If[set==3 && datanum==9, topname=StringJoin["Synthetic data 3"]];

If[set==3 && datanum==10, topname=StringJoin["Synthetic data 3"]];

If[set==3 && datanum==11, topname=StringJoin["Synthetic data 4"]];

If[set==3 && datanum==12, topname=StringJoin["Synthetic data 4"]];

Return[topname]]

End[]

EndPackage[]

PACKAGE 8

(*:Title:: overj *)

(*:Summary:: SOF *)

(*:References:: *)

(*Date 2024 Stuart G. Baker *)

BeginPackage["overjsim`"]

GenSimE::usage ="GenData[datanum]"

GenSimW::usage ="GenData[datanum]"

GenSimW1::usage ="GenData[datanum]"

Clear[GenSimE,GenSimW,GenSimW1]

Begin["Private`"]

GenSimE[adj_,p_,mean_,n_,agevec_,show_:False]:=GenSimCore[adj,p,{0,mean},n,"E",show,agevec]

GenSimW[adj_,p_,mean_,n_,agevec_,show_:False]:=GenSimCore[adj,p,{1.5,mean},n,"W",show,agevec]

GenSimW1[adj_,p_,{w1_,mean_},n_,agevec_,show_:False]:=GenSimCore[adj,p,{w1,mean},n,"W",show,agevec]

GenSimCore[adj_,p_,{w1_,mean_},n_,dtype_,showplot_,agevec_]:=

Module[{x,w,z,y, pair, range, tri, plot, plotx,sof,mat0,mat,meanpdfr,meanpdf,a0,b0,g},

a0=Min[agevec];

b0=Max[agevec];

(*time enter state*)

z = RandomVariate[TriangularDistribution[{a0-5, b0},b0], n];

(*sojourn time*)

If[dtype=="E",

meanpdf=Mean[ExponentialDistribution[1/mean]];

w=RandomVariate[ExponentialDistribution[1/mean],n]];

If[dtype=="W",

meanpdf=Mean[WeibullDistribution[w1, mean]];

w=RandomVariate[WeibullDistribution[w1, mean],n]];

(*indicator of indolent state*)

g=RandomVariate[BernoulliDistribution[p],n];

(*competing risk death age*)

c= RandomVariate[TriangularDistribution[{a0-5, b0+30},b0+30], n];

(*age at symptomatic cancer if no competing risk*)

y = z+ (1-g) w + g (100-z);

(*Indicator of overdiagnosis based on competing mortality*)

paircy=Transpose[{z,y,c}];

o=GenZ[#]& /@ paircy;

(*compute cross-sectional data*)

pairvec=Transpose[{z,y,c}];

mat0=GenAge[pairvec,#]& /@ agevec;

mat=Drop[mat0,-1];

(*estimated SOF in simulation*)

sof=GenSimSOF[{z,y,o},agevec,showplot];

Return[{mat,sof,meanpdf}]]

GenZ[{z_,y_,c_}]:=

Module[{o},

If[c < y && c>z ,o=1, o=0];

Return[o]]

GenAgeLine[a_,n_]:=Graphics@Line[{{a,0},{a,n}}];

GenLine[{score_,r_,z_,y_}]:= Graphics@Line[{{z,r},{y,r}}];

GenAge[pairvec_,a_]:=

Module[{xf,xi,xs,res },

xf=Length@Select[pairvec,(#[[1]]< a && #[[2]] >= a && #[[3]]>a)&];

xi=Length@Select[pairvec,(#[[1]]> a && #[[2]] < (a+1)&& #[[3]]>a+1)&];

xs=Length@Select[pairvec,(#[[1]]> a && #[[1]] < (a+1) && #[[2]] >= (a+1)&& #[[3]]>a+1)&];

res={a,xf,xi,xs};

Return[res]]

(*Compute estiamted SOF in simulation*)

GenSimSOF[{x_,y_,o_},agevec_,showplot_:False]:=

Module[{trivec,mat,a,xf0,xf1,xs0,xs1,ns0,ns1,nf0,nf1,SOF},

trivec=Transpose[{x,y,o}];

mat=GenAgeSOF[trivec,#]& /@ agevec;

{a,xf0,xf1,xs0,xs1,xi}=Transpose[mat];

ni=Total[xi];

nz1=Total[z];

nf0=xf0[[1]];

nf1=xf1[[1]];

ns1=Total[xs1];

ns0=Total[xs0];

SOF=(nf1+ns1)/(nf1+nf0+ns1+ns0)//N;

len=Length[z];

SOFZ=Total[z]/(len-ni)//N;

Return[SOF]]

GenAgeSOF[trivec_,a_]:=

Module[{xf0,xf1,xs0,xs1,res,xi },

xf0=Length@Select[trivec,(#[[1]]< a && #[[2]] >= a && #[[3]]==0)&];

xf1=Length@Select[trivec,(#[[1]]< a && #[[2]] >= a && #[[3]]==1)&];

xs0=Length@Select[trivec,(#[[1]]> a && #[[1]] < (a+1) && #[[2]] >= (a+1) && #[[3]]==0)&];

xs1=Length@Select[trivec,(#[[1]]> a && #[[1]] < (a+1) && #[[2]] >= (a+1) && #[[3]]==1)&];

xi=Length@Select[trivec,(#[[1]]> a && #[[2]] <( a+1))&];

(*OVER SET OF AGES*)

res={a,xf0,xf1,xs0,xs1,xi};

Return[res]]

PositiveSign[x_] := Sign[Sign[x] - 1] + 1

NegativeSign[x_] := 1 - Sign[Sign[x] + 1]

End[]

EndPackage[]

PACKAGE 9

(*:Title:: overj *)

(*:Summary:: SOF *)

(*:References:: *)

(*Date 2024 Stuart G. Baker *)

BeginPackage["overjdataSEI`","overjsim`"]

GenSEIDataSimE::usage ="GenSEIDataMayo[]"

GenSEIDataSimW::usage ="GenSEIDataMayo[]"

GenSEIDataSimW1::usage ="GenSEIDataMayo[]"

GenSEIDataMayo::usage ="GenSEIDataMayo[]"

GenSEIDataPLCOL::usage="GenSEIDataPLOCP[]"

GenSEIDataNLSTC::usage="GenSEIDataPLOCP[]"

GenSEIDataNLSTX::usage="GenSEIDataPLOCP[]"

Clear[GenSEIDataSimE,GenSEIDataSimW,GenSEIDataSimW1,

GenSEIDataMayo,GenSEIDataPLOCOL,

GenSEIDataNLSTC, GenSEIDataNLSTX]

Begin["Private`"]

GenSEIDataSimE[adj_,p_,mean_,n_,show_,agennum_]:=GenSEIDataSim[adj,p,{0,mean},"E",n,show,agevec];

GenSEIDataSimW[adj_,p_,mean_,n_,show_,agevec_]:=GenSEIDataSim[adj,p,{1.5,mean},"W",n,show,agevec];

GenSEIDataSimW1[adj_,p_,{w1_,mean_},n_,show_,agevec_]:=GenSEIDataSim[adj,p,{w1,mean},"W1",n,show,agevec];

GenSEIDataSim[adj_,p_,{w1_,mean_},dtype_,n_,show_,agevec_]:=

Module[{matz,mat,obs,age,numint0,int0,numscr0,scr0,numint1,

int1,numscr1,scr1,numint2,int2,numscr2,scr2,numint3,int3,numscr3,scr3,

ni,ns,nf,xi,xs,xf,xf0,xs0,name,res,sofs,sof},

(*One data set for the mean*)

If[dtype=="E", {mat,sof,meanpdf}=GenSimE[adj,p,mean,n,agevec]];

If[dtype=="W", {mat,sof,meanpdf}=GenSimW[adj,p,mean,n,agevec]];

If[dtype=="W1", {mat,sof,meanpdf}=GenSimW1[adj,p,{w1,mean},n,agevec]];

{age,xf,xi,xs}=Transpose[mat];

len= Length[age];

size=10000//N;

nf=Table[size,len];

ni=nf;

ns=nf;

sofs=ToString@Round[sof,.01];

name=StringJoin[dtype," OP = ",ToString[p],"; MST=",ToString[meanpdf],"; SOF= ",sofs];

(*DATA SHOW*)

If[show,

Print[name];

mat=Transpose[{age,xf,nf,xs,ns}];

colname={"age","xf","nf","xs","ns"};

Print["Total xi ni ",{Total[xi],Total[ni]}];

Print["Total xs ns ",{Total[xs],Total[ns]}];

Print@TableForm[mat,TableHeadings->{None,colname}]];

res=GenSet[name,{age,xf,xi,xs,nf,ni,ns},"B"];

ps=ToString[p];

meanpdfs=ToString[meanpdf];

sofs=ToString[Round[sof,.01]];

Return[{res,p,meanpdf,sof}]]

GenSEIDataPLCOL[adj_,show_:False]:=

Module[{matz,mat,obs,age,numint0,int0,numscr0,scr0,numint1,int1,numscr1,scr1,numint2,int2,numscr2,scr2,numint3,int3,numscr3,scr3,

ni,ns,nf,xi,xs,xf,name,res},

mat0 = ReadList["ozdataplcol.m", Number, RecordLists -> True];

mat=Select[mat0,(#[[2]]<=75)&];

If[adj==1, matz=Drop[mat,10], matz=mat];

{obs,age,numint0,int0,numscr0,scr0,numint1,int1,numscr1,scr1,numint2,int2,numscr2,scr2}=Transpose[matz];

ni=numint0+numint1+numint2;

ns=numscr1+numscr2;

nf=numscr0;

xi=int0+int1+int2;

xs=scr1+scr2;

xf=scr0;

name="PLCO lung cancer: x-ray (1993)";

name="PLCO x-ray (1993)";

If[show,

Print[name];

mat=Transpose[{age,xf,nf,xs,ns}];

colname={"age","xf","nf","xs","ns"};

Print["Total xi ni ",{Total[xi],Total[ni]}];

Print["Total xs ns ",{Total[xs],Total[ns]}];

Print@TableForm[mat,TableHeadings->{None,colname}]];

res=GenSet[name,{age,xf,xi,xs,nf,ni,ns},"B"];

Return[res]]

GenSEIDataNLSTC[adj_,show_:False]:=

Module[{mat,obs,age,numint0,int0,numscr0,scr0,numint1,int1,numscr1,scr1,numint2,int2,numscr2,scr2,numint3,int3,numscr3,scr3,

ni,ns,nf,xi,xs,xf,name,res},

mat0 = ReadList["ozdataNLSTct.m", Number, RecordLists -> True];

mat=Select[mat0,(#[[2]]<=74)&];

If[adj==1, matz=Drop[mat,10], matz=mat];

{obs, age,numint0,numint1,numint2,int0,int1,int2,numscr0,numscr1, numscr2,scr0,scr1,scr2}= Transpose[matz];

ni=numint0+numint1+numint2;

ns=numscr1+numscr2;

nf=numscr0;

xi=int0+int1+int2;

xs=scr1+scr2;

xf=scr0;

name="NLST lung cancer: CT (2002)";

name="NLST CT (2002)";

If[show,

Print[name];

mat=Transpose[{age,xf,nf,xs,ns}];

colname={"age","xf","nf","xs","ns"};

Print["Total xi ni ",{Total[xi],Total[ni]}];

Print["Total xs ns ",{Total[xs],Total[ns]}];

Print@TableForm[mat,TableHeadings->{None,colname}]];

res=GenSet[name,{age,xf,xi,xs,nf,ni,ns},"B"];

Return[res]]

GenSEIDataNLSTX[adj_,show_:False]:=

Module[{mat,obs,age,numint0,int0,numscr0,scr0,numint1,int1,numscr1,scr1,numint2,int2,numscr2,scr2,numint3,int3,numscr3,scr3,

ni,ns,nf,xi,xs,xf,name,res},

mat0 = ReadList["ozdataNLSTxray.m", Number, RecordLists -> True];

mat=Select[mat0,(#[[2]]<=74)&];

If[adj==1, matz=Drop[mat,10], matz=mat];

{obs, age,numint0,numint1,numint2,int0,int1,int2,numscr0,numscr1, numscr2,scr0,scr1,scr2}= Transpose[matz];

ni=numint0+numint1+numint2;

ns=numscr1+numscr2;

nf=numscr0;

xi=int0+int1+int2;

xs=scr1+scr2;

xf=scr0;

name="NLST lung cancer: x-ray (2002)";

name="NLST x-ray (2002)";

If[show,

Print[name];

mat=Transpose[{age,xf,nf,xs,ns}];

colname={"age","xf","nf","xs","ns"};

Print["Total xi ni ",{Total[xi],Total[ni]}];

Print["Total xs ns ",{Total[xs],Total[ns]}];

Print@TableForm[mat,TableHeadings->{None,colname}]];

res=GenSet[name,{age,xf,xi,xs,nf,ni,ns},"B"];

Return[res]]

GenSEIDataMayo[adj_,show_:False]:=

Module[{mat,age,pf,pi,ps,pr,pc,nf,ni,ns,nr,nc,k0,s0,h0,avec, ageadd,nvec, datavec,res},

mat=

{{51, 2, 1, 1, 1, 0, 1, 0, 375, 3086, 2363, 127, 54, 1928, 706},

{52, 5, 1, 2, 0, 0, 2, 0, 392, 3147, 2444, 162, 88, 1979, 1264},

{53, 4, 0, 3, 0, 0, 0, 1, 365, 3113, 2414, 142, 117, 1978, 1732},

{54, 4, 1, 1, 0, 1, 0, 1, 374, 3125, 2417, 146, 114, 1966, 1684},

{55, 5, 2, 4, 0, 0, 1, 4, 354, 3106, 2419, 132, 139, 1921, 1736},

{56, 4, 1, 2, 1, 0, 4, 2, 364, 3126, 2414, 130, 122, 1910, 1722},

{57, 3, 0, 6, 1, 0, 2, 1, 366, 3100, 2417, 106, 136, 1891, 1663},

{58, 5, 0, 4, 0, 0, 2, 3, 317, 2985, 2353, 78, 110, 1834, 1664},

{59, 4, 1, 3, 0, 1, 2, 1, 330, 3005, 2385, 72, 101, 1785, 1641},

{60, 4, 2, 5, 0, 0, 1,10, 315, 2933, 2330, 72, 74, 1773, 1601},

{61, 5, 0, 4, 1, 0, 3, 2, 301, 2836, 2275, 74, 61, 1692, 1526},

{62, 5, 1, 2, 0, 0, 2, 3, 302, 2742, 2162, 79, 57, 1647, 1485},

{63, 5, 0, 3, 0, 0, 4, 5, 248, 2563, 2072, 86, 60, 1499, 1504},

{64, 6, 2, 9, 0, 1, 5, 3, 242, 2410, 1903, 77, 59, 1447, 1389},

{65, 3, 4, 2, 1, 1, 4, 5, 232, 2224, 1768, 61, 65, 1409, 1355},

{66, 5, 1, 2, 0, 0, 1, 0, 170, 2004, 1612, 39, 65, 1213, 1272},

{67, 3, 1, 6, 0, 0, 3, 2, 176, 1834, 1446, 49, 64, 1134, 1176},

{68, 5, 3, 3, 0, 0, 2, 2, 149, 1550, 1213, 57, 54, 974, 1099},

{69, 3, 0, 5, 1, 0, 2, 3, 124, 1357, 1068, 61, 29, 904, 963}};

(* AGE F I S R3 R6 C3 C6 NF NI NSX NR3 NR6 NC3 NC6 *)

If[adj==1, matz=Drop[mat,10], matz=mat];

{age,xf,xi,xs,xr3,xr6,xc3,xc6,nf,ni,ns,mr3,mr6,mc3,mc6}=Transpose[matz];

res=GenSet[name,{age,xf,xi,xs,nf,ni,ns},"B"];

name="MLP cytology + x-ray (1971)";

If[show,

Print[name];

mat=Transpose[{age,xf,nf,xs,ns}];

colname={"age","xf","nf","xs","ns"};

Print["Total xi ni ",{Total[xi],Total[ni]}];

Print["Total xs ns ",{Total[xs],Total[ns]}];

Print@TableForm[mat,TableHeadings->{None,colname}]];

res=GenSet[name,{age,xf,xi,xs,nf,ni,ns},"B"];

Return[res]]

GenSet[name_,datavec_,survtype_]:=

Module[{datavec0,age,xf,xi,xs,nf,ni,ns,nF,nS,xS,xI,Favg1,Savg1,k,Fvec,Svec,Favg0,Savg0,nvecS,nvecF,res},

datavec0= Select[Transpose[datavec],(#[[1]]<=70)&];

{age,xf,xi,xs,nf,ni,ns}=Transpose[datavec0];

nF=Total[nf];

nS=Total[ns];

nI=Total[ni];

xS=Total[xs];

xI=Total[xi];

Favg1=Total[xf]/nF//N;

Savg1=Total[xs]/nS//N;

k=Length[age];

Fvec=xf/nf//N;

Svec=xs/ns//N;

Favg0=Mean[Fvec]//N;

Savg0=Mean[Svec]//N;

nvecS=ns;

nvecF=nf;

res={name,age,Favg0,Savg0,Favg1,Savg1,nF,nS, nI,xS,xI, k,Fvec,Svec,nvecF,nvecS,survtype};

Return[res]]

End[]

EndPackage[]PACKAGE 10

(*:Title:: overj *)

(*:Summary:: *)

(*:References:: *)

(*Date 2024 Stuart G. Baker *)

BeginPackage["overjdataRCT`"]

OverLungRCT::usage ="GenRCTDataMayo[]"

Clear[OverLungRCT]

Begin["Private`"]

OverLungRCT[]:=

Module[{dataRCT1,matRCT1,dataRCT2,matRCT2},

dataRCT1=GenRCTDataMayo[];

matRCT1=OverDiagRCT[dataRCT1];

Print["MAYO"];

Print@TableForm[matRCT1];

dataRCT2=GenRCTDataPLCOL[];

matRCT2=OverDiagRCT[dataRCT2];

Print["PLCO"];

Print@TableForm[matRCT2];

Return[Null]]

OverDiagRCT[mat_,show_:True]:=OverDiagRCTCore[#,show]& /@ mat

OverDiagRCTCore[{name_,time_,nc_,ns_,n0_,r0_,f_},show_]:=

Module[{SOF,SOFr,seSOFr,r,var,se,ser},

n1=nc+ns;

SOF=Max[0,(n1-n0)/(ns f)//N];

SOF=(n1-n0)/(ns f)//N;

var=((n0-nc)^2 + (n0+nc) ns) /(f^2 ns^3)//N;

var0=(nc^2 + (n0+ns) n0 +nc (ns- 2 n0)) /(f^2 ns^3)//N;

se=Sqrt[var];

If[show,

Print[" name time, n0 n1 ns ",{name,time, n0,n1,ns}]];

SOFr=Round[SOF,.01];

ser=Round[se,.01];

(*One sided if less than zero*)

If[SOF>=0,SOFlow=Max[0,SOF-1.96se], SOFlow=SOF];

SOFupp=Min[1,SOF+1.96se];

SOFlowr=Round[SOFlow,.01];

SOFuppr=Round[SOFupp,.01];

vec={"RCT",time,SOFr,SOFlowr,SOFuppr};

Return[vec]]

GenRCTDataMayo[]:=GenRCTDataMayo[#]& /@{10,15,20};

GenRCTDataMayo[time_]:=

Module[{nc,ns,n0,r,name,timex},

ns=143;

If[time==10, n0=204; n1=256];

If[time==15, n0=276; n1=338];

If[time==20, n0=360; n1=425];

nc=n1-ns;

r=1;

name="Mayo";

f=.93;

(*since randomization*)

timex=time;

type=StringJoin[ToString[timex], " YSR"];

Return[{name,type,nc,ns,n0,r,f}]]

GenRCTDataPLCOL[]:=GenRCTDataPLCOL[#]& /@{12, 16};

GenRCTDataPLCOL[time_]:=

Module[{mat,vtime, v0,v1,vs,n0,n1,ns,nc,res,yr},

mat=

{{1,0,109,183,122},

{2,1,125,124,54},

{3,2,143,140,62},

{4,3,127,143,72},

{5,4,151,108,0},

{6,5,138,121,0},

{7,6,134,174,0},

{8,7,166,150,0},

{9,8,156,156,0},

{10,9,144,157,0},

{11,10,129,160,0},

{12,11,124,107,0},

{13,12,83,92,0},

{14,13,58,55,0},

{15,14,27,24,0},

{16,15,2,13,0}};

{vtime, yr, v0,v1,vs}=Transpose[mat];

n0=Total@Take[v0, time];

n1=Total@Take[v1, time];

ns=Total@Take[vs,time];

nc=n1-ns;

r=1;

name="PLCO-L";

f=.89;

type=StringJoin[ToString[time], " YSR"];

res={name,type,nc,ns,n0,r,f};

Return[res]]

End[]

EndPackage[]

PACKAGE 11

(*:Title:: overj *)

(*:Summary:: SOF *)

(*:References:: *)

(*Date 2024 Stuart G. Baker *)

BeginPackage["overjfig`"]

PlotFig1::usage="PlotFig1"

PlotFigS1::usage="PlotS2"

PlotFigS2::usage="PlotS2"

Clear[PlotFig1,PlotFigS1,PlotFigS2]

Begin["Private`"]

PlotFig1[]:=

Module[{max,parset0,parset,qq,

n,range,range0,xvec,yvec,triset,

agevec,linevec,hlines,vlines,plotx,i,j},

(*SDPC states*)

max=5;

pairset0=Partition[Flatten@Table[{Adj[i], Min[ i + j, max]}, {i, 0, max}, {j, .5,max+1 - i}], 2];

pairset=IntervalCase[#,max]& /@ Drop[Drop[pairset0,-3],1];

qq=-2.5;

pairset=

{{qq,1.5},

{qq,2.5},

{qq,3.5},

{qq,4.5},

{qq,6},

{1.25,1.5},

{1.25,2.5},

{1.25,3.5},

{1.25,4.5},

{1.25,6},

{2.25,2.5},

{2.25,3.5},

{2.25,4.5},

{2.25,6},

{3.25,3.5},

{3.25,4.5},

{3.25,6}};

n=Length[pairset];

range=Range[n];

range0=AdjY[#]& /@ range;

{xvec,yvec}=Transpose[pairset];

triset=Transpose[{xvec,yvec,range0}];

hlines=GenHLine[#]& /@ triset;

(*SCREEN LINES*)

vlines=GenVLine[#,Max[range0]]&/@{-1,2,3,4, 7};

(*RIGHT TICKS*)

nameR=GenNameRight[#,max-1,max]& /@ triset;

tickR0=Transpose[{range0, nameR}];

dots=Rotate[Style["...",{24,Black,Bold}], 90 Degree];

tickR=tickR0~Join~ {{7,dots}};

(*LABEL BELOW*)

hname={"a",Style["...",{24,Black,Bold}], "68","69","70","100"};

rangeB={-1,0.5, 2, 3, 4, 7};

tickB=Transpose[{rangeB,hname}];

Ls="(";

Rs=")";

dots=Style["...",{24,Black,Bold}];

as="50";

cs="100";

Fs="F";

bs="70";

bs1="69";

b2"68";

Ss="S";

Hs="H";

Ds="D";

tots="Total = D";

us ="\n";

cms=",";

m1s="-1";

m2s="-2";

h1=rz@StringJoin[as,us,Fs,Ls,as,Rs];

h2=dots;

h3=rz@StringJoin["68",us,Ss,Ls,"68",Rs];

h4=rz@StringJoin["69",us,Ss,Ls,"69",Rs];

h5=rz@StringJoin["70",us,Ss,Ls,"70",Rs];

h6=rz@StringJoin[cs,us,tots,Ls,as,cms,bs,Rs];

hname={"a\nF(a)",Style["...",{24,Black,Bold}], "b-2\nS(b-2)","b-1\nS(b-1)","b\nS(b)","c\nTotal=D(a,b) "};

h0="Cohort 1";

hname={h0,h1,h2,h3,h4,h5,h6};

rangeB={-3,-1,0.5, 2, 3, 4, 7};

tickB=Transpose[{rangeB,hname}];

(*LABEL TOP*)

tickT={{-3,"Cohort 2"},{0, rz@"receives first screen at age 70"},{4,rz@"F(70)"}};

(*POINTS*)

points=GenMPoint[#,max-1]& /@ triset;

(*bottom name*)

k="\!\(\[Lambda]\)";

nameB=Style[StringJoin["Total = H(",k,")"], 14,Bold, Black];

nameB=rz@StringJoin["Total = H(",k,")"];

textB=Graphics@Text[nameB,{7.82,0},{0,0}];

nameS=Style["Total = D(50,70)", 12,Bold, Black];

textS=Graphics@Text[nameS,{8,-1},{-1,0}];

lab=GenLabR[#]& /@ triset;

(*FINAL PLOT*)

plotx=Show[hlines,vlines,points,textB,lab,AspectRatio->1, PlotRange->{{-3, max+2},Automatic},

LabelStyle->{12,Black,Bold},FrameTicks->{{None, tickR},{tickB,tickT}},Frame->True,ImageSize->Large];

Return[plotx]];

rz[x_]:=Style[x,12,Italic,Black, Bold]

sz[x_]:=Style[x,14,Black, Bold]

AdjY[y_]:=

Module[{res},

If[y>5, res=y+3, res=y];

Return[res]]

Adj[i_]:=

Module[{res},

If[i>0, res=i+.25, res=-2];

Return[res]]

GenMPoint[{x_,y_,z_},m_]:=

Module[{res},

If[y>= m && y< m+1,

res=Graphics@{PointSize[.03], Darker[Green],Point[{Max[-1,Ceiling[x]],z}]},

res={}];

Return[res]]

GenLabR[{x_,y_,j_}]:=

Module[{res},

If[y==6,

res= Graphics@Text[Style["R",12, Black, Bold],{y,j},{-1,0}],

res= Graphics@Text[Style["C",12, Black, Bold],{y,j},{-1,0}]];

Return[res]]

GenNameRight[{x_,y_,z_},m_,max_]:=

Module[{k,res,maxs,ys,name},

k="\!\(\[Lambda]\)";

If[y>= m && y< m+1 && x >3,

name=StringJoin["S(70) G(70;",k,")"]];

If[y>= m && y< m+1 && x >2 && x<3,

name=StringJoin["S(69) G(69; ",k,")"]];

If[y>= m && y< m+1 && x >1 && x< 2,

name=StringJoin["S(68) G(68; ",k,")"]];

If[y>= m && y< m+1 && x <1,

name=StringJoin["F(50) G(50; ",k,")"]];

If[y< m || y>=m+2, name=" "];

(*If[y== max+2, name="Overdiagnosed"]; *)

res=Graphics@Text[Style[name,{14,Black,Bold}], {m+1.2, z},{1,0}];

res=Style[name,{12,Italic,Black,Bold}];

Return[res]]

GenNameRightAB[{x_,y_,z_},m_,max_]:=

Module[{k,res,maxs,ys,name},

k="\!\(\[Lambda]\)";

If[y>= m && y< m+1 && x >3,

name=StringJoin["S(b) {1-SOF(",k,")} G(b;",k,")"]];

If[y>= m && y< m+1 && x >2 && x<3,

name=StringJoin["S(b-1) {1-SOF(",k,")} G(b-1; ",k,")"]];

If[y>= m && y< m+1 && x >1 && x< 2,

name=StringJoin["S(b-2) {1-SOF(",k,")} G(b-2; ",k,")"]];

If[y>= m && y< m+1 && x <1,

name=StringJoin["F(a) {1-SOF(",k,")} G(a; ",k,")"]];

If[y< m || y>=m+2, name=" "];

(*If[y== max+2, name="Overdiagnosed"]; *)

res=Graphics@Text[Style[name,{14,Black,Bold}], {m+1.2, z},{1,0}];

res=Style[name,{12,Italic,Black,Bold}];

Return[res]]

IntervalCase[{x_,y_},max_]:=

Module[{x0,y0},

If[x==y, x0=x-.25; y0=y+.25, x0=x; y0=y];

(*expand*)

If[y>=max,y0=max+2];

Return[{x0,y0}]]

GenHLine[{x_,y_,z_}]:=Graphics@{Dashing[{.01,.01}],Line[{{x,z},{y,z}}]}

GenVLine[a_,n_]:= Graphics@Line[{{a,0},{a,n}}]

PlotFigS1[]:=

Module[{x,dI,dS,r,rtrue,plot,name1,name2,text1,text2,plotx},

h=1/m;

dI=Integrate[1 - Exp[-h(1-x)], {x, 0, 1}];

dS=Integrate[Exp[-h(1- x)], {x, 0, 1}];

r=dI/dS;

rtrue=dI/(dS+.2);

plot=Plot[{r,rtrue}, {m,.01,3}, PlotStyle->{Purple,{Dashing[{.01,.01}],Orange}},

PlotLabels->Placed[{"R","R*"},Left]];

name1="Bound on ratio";

name2="True ratio";

plotx=Show[plot,Frame->True,FrameLabel->{"MST", "Ratio"},ImageSize->Medium];

Print[plotx];

Return[Null]]

PlotFigS2[]:=

Module[{mean1,mean2,plot,dash},

dash=Dashing[{.01,.01}];

plot=Plot[{

PDF[ExponentialDistribution[1],x],

PDF[WeibullDistribution[1.5, 1],x],

PDF[ExponentialDistribution[1/2],x],

PDF[WeibullDistribution[1.5, 3],x]},

{x,0,4}, PlotRange->All, PlotStyle->{Red, {dash,Purple}, Orange, {dash,Blue}},

PlotLabels->{

Style["Exponential(1)",Red,Bold],

Style["Weibull(1.5,1)",Purple,Bold],

Style["Exponential(2)",Orange,Bold],

Style["Weibull(1.5,3)",Blue,Bold]}];

Print@Show[plot, Frame->True, LabelStyle->{Bold,Black},

FrameLabel->{"Sojourn time",None}, PlotRange->All,ImageSize->Medium];

Return[Null]]

End[]

EndPackage[]
